# Efficacy and safety of add‐on antiseizure medications for focal epilepsy: A network meta‐analysis

**DOI:** 10.1002/epi4.12997

**Published:** 2024-06-18

**Authors:** Hesheng Zhang, Zhujing Ou, Enhui Zhang, Wenyu Liu, Nanya Hao, Yujie Chen, Yutong Liu, Hui Ye, Dong Zhou, Xintong Wu

**Affiliations:** ^1^ Neurology Department West China Hospital of Sichuan University Chengdu Sichuan China; ^2^ Ignis Therapeutics (Shanghai) Limited Shanghai China

**Keywords:** add‐on therapy, antiseizure medication, focal epilepsy, network meta‐analysis

## Abstract

**Objective:**

Several antiseizure medications (ASMs) have been approved for the treatment of focal epilepsy. However, there is a paucity of evidence on direct comparison of ASMs. We evaluated the comparative efficacy and safety of all approved add‐on ASMs for the treatment of focal epilepsy using network meta‐analysis.

**Methods:**

Data through extensive literature search was retrieved from PubMed, Embase, Cochrane, and ClinicalTrial.gov databases using predefined search terms from inception through March 2023. PRISMA reporting guidelines (CRD42023403450) were followed in this study. Efficacy outcomes assessed were ≥50%, ≥75%, and 100% responder rates. Patient retention rate and safety outcomes such as overall treatment‐emergent adverse events (TEAEs) and individual TEAEs were assessed. “Gemtc” 4.0.4 package was used to perform Bayesian analysis. Outcomes are reported as relative risks (RRs) and 95% confidence interval (CI).

**Results:**

Literature search retrieved 5807 studies of which, 75 studies were included in the analysis. All ASMs showed significantly higher ≥50% responder rate compared with placebo. Except the ≥75% seizure frequency reduction for zonisamide (2.23; 95% CI: 1.00–5.70) and 100% for rufinamide (2.03; 95% CI: 0.54–11.00), all other interventions showed significantly higher ≥75% and 100% responder rates compared with placebo. Among treatments, significantly higher 100% responder rate was observed with cenobamate compared to eslicarbazepine (10.71; 95% CI: 1.56–323.9) and zonisamide (10.63; 95% CI: 1.37–261.2). All ASMs showed a lower patient retention rate compared to placebo, with the least significant value observed for oxcarbazepine (0.77; 95% CI: 0.7–0.84). Levetiracetam showed a lower risk of incidence (1.0; 95%CI: 0.94–1.1; SUCRA: 0.885067) for overall TEAE compared with other medications.

**Significance:**

All approved ASMs were effective as add‐on treatment for focal epilepsy. Of the ASMs included, cenobamate had the greatest likelihood of allowing patients to attain seizure freedom.

**Plain Language Summary:**

This article compares the efficacy and safety of antiseizure medications (ASMs) currently available to neurologists in the treatment of epileptic patients. Several newer generation ASMs that have been developed may be as effective or better than the older medications. We included 75 studies in the analysis. In comparison, all drugs improved ≥50%, ≥75% and 100% responder rates compared to control, except for Zonisamide and Rufinamide in the ≥75% and 100% responder rate categories. Retention of patients undergoing treatment was lower in drugs than placebo. All drugs were tolerated, the levetiracetam showed the best tolerability. Cenobamate more likely help completely to reduce seizures.

**Key points**

- All add‐on antiseizure medications (ASMs) approved for the treatment of epilepsy showed that they were effective compared to placebo.
- All ASMs showed higher ≥50% responder rate, while most of the ASMs showed higher ≥75% and 100% responder rates compared with placebo.
- Patient retention rates were lower for all ASMs compared to placebo.
- All ASMs showed the acceptable tolerability, placebo and LEV have the lowest risk.
- Cenobamate had the greatest likelihood of allowing patients to attain seizure freedom.

## 1 INTRODUCTION

Epilepsy is one of the most common neurologic diseases, characterized by recurrent and transient episodes of excessive neurological activity, affecting more than 50 million individuals worldwide.^1^, ^2^, ^3^ Focal epilepsy is the predominant type (accounting for 60%) which occurs when seizures have their onset within networks limited to one hemisphere.^4^, ^5^, ^6^, ^7^ The current prescribed therapy to treat epilepsy antiseizure medication (ASM) which provides symptomatic relief, therefore, minimizes treatment‐related adverse effects and restores quality of life (QoL).^8^ Despite advantageous, one‐third of patients with epilepsy are unresponsive to ASMs or other treatments resulting in drug‐resistant epilepsy.^9^, ^10^ The incidence of drug‐resistant epilepsy reported by a meta‐analysis study in 2021 was 13.7% (95% CI: 9.2–19.0) in population/community‐based populations and 36.3% (95% CI: 30.4–42.4) in the clinic‐based cohorts.^11^ Drug‐resistant epilepsy and patients who are unable to obtain sustained seizure freedom after a trial of two ASMs may require combination therapy for effective disease management.

ASMs approved by the Food and Drug Administration (FDA) and European Medicines Agency (EMA) as adjunctive therapies for focal epilepsy included first‐generation ASMs ‐ valproate (VPA) and carbamazepine (CBZ); second‐generation drugs ‐ gabapentin (GBP), lamotrigine (LTG), levetiracetam (LEV), oxcarbazepine (OXC), pregabalin (PGB), tiagabine (TGB), topiramate (TPM), vigabatrin (VGB), and zonisamide (ZNS); and third‐generation ASMs ‐ eslicarbazepine (ESL), lacosamide (LCM), perampanel (PER), brivaracetam (BRV), rufinamide (RUF), retigabine (ezogabine, EZG), and cenobamate (CNB). Appropriate ASM should be chosen based on many factors such as efficacy, safety, cost, and availability. However, there're very limited head‐to‐head trials comparing efficacy and safety of different ASMs. Therefore, indirect comparison of treatments by network meta‐analysis (NMA) is an alternative way to understand the comparative effectiveness of approved treatments.

To date, only a few NMAs have been published for the treatment of ASMs in focal epilepsy, either with comparison of limited numbers of ASMs or insufficient indicators/endpoints analyzed.^2^, ^12^, ^13^, ^14^, ^15^ Thus, to provide an up‐to‐date and referable evidence for the clinical decision‐making, we assessed the comparative efficacy and safety of approved ASMs as add‐on treatment for focal epilepsy in adolescent and adult patients by performing a NMA of the existing randomized controlled trials (RCTs). To our knowledge, this is the first study to compare all three generations of ASMs that are approved to date.

## 2 MATERIALS AND METHODS

In this NMA, findings are reported following the Preferred Reporting Items for Systematic reviews and Meta‐Analyses (PRISMA) extension guideline.^16^ The study protocol has been registered in the International Prospective Register of Systematic Reviews (PROSPERO) database (CRD42023403450).

### 2.1 Data sources and search strategy

PubMed, Embase, Cochrane, and ClinicalTrial.gov databases were systematically searched for articles published from inception until March 2023 by using the following search terms: “focal epilepsy,” “partial epilepsy,” “lacosamide,” “eslicarbazepine,” “perampanel,” “brivaracetam,” “cenobamate,” “pregabalin,” “tiagabine,” “vigabatrin,” “zonisamide,” “levetiracetam,” “oxcarbazepine,” “topiramate,” “lamotrigine,” “carbamazepine,” “valproate,” “rufinamide,” “gabapentin,” “retigabine,” and “randomised controlled trial.” The study selection followed three steps. First, two reviewers independently screened the title of studies. Second, two reviewers screened and selected abstracts independently and compared results. Disagreements were resolved by a third reviewer. Third, two reviewers independently examined the full text of articles to confirm inclusion and the clinical similarity. Disagreements were resolved by consensus or, by a third reviewer, if necessary.

### 2.2 Inclusion and exclusion criteria

Studies were included if they met the following criteria: (1) Randomized, double‐blinded controlled, parallel group, add‐on studies; (2) adolescent and adult patients (aged ≥12 years) with focal epilepsy or refractory focal epilepsy or drug‐resistant focal epilepsy; (3) patients receiving add‐on ASMs with any of the marketed drugs for epilepsy that includes VPA, CBZ, GBP, LTG, LEV, OXC, PGB, TGB, TPM, VGB, ZNS, ESL, LCM, PER, BRV, RUF, EZG, and CNB; (4) reporting at least one of the following clinical outcomes: ≥50% responder rate, ≥75% responder rate, and seizure freedom from baseline seizure frequency and adverse events (AEs); (5) only maintenance doses of the aforementioned drugs approved by the FDA and EMA; and (6) studies that included a prospective baseline period of at least 4 weeks with a minimum 8‐week maintenance treatment period or periods of stable dosing for dose escalation studies.

Case reports, open‐label studies, reviews, systematic reviews, meta‐analysis, noncomparative studies, and cost‐related studies were excluded. In addition, duplicated publications from the same patient population studies and studies published in language other than English were also excluded.

### 2.3 Data extraction and study outcomes

Data including author, year of publication, title, study design, demographics of the study population, and outcomes of interest were extracted by two independent reviewers from the included studies into standardized MS Office Excel sheet. In studies involving multiple arms with different doses, the events and number of patients in different dose groups were pooled and considered as one arm for analysis. For multiple publications on the same patient population, both the first report and the most recent publication were selected and the most recent data were used for the analysis.

The primary efficacy outcomes were the proportion of participants achieving response rates of ≥50%, ≥75%, and seizure freedom from baseline seizure frequency, whereas the secondary outcome was patient retention rate. Safety was assessed by treatment‐emergent adverse events (TEAEs), defined as any AE with the onset during or after the study drug administration during the study period. Further, TEAEs were reported as central nervous system (CNS)‐related TEAEs and non‐CNS‐related TEAEs. The TEAE incidences associated with CNS were grouped according to a previous meta‐analysis study on CNS‐related TEAEs related to ASMs. The CNS‐related TEAEs were categorized into five broad classes, those affecting vigilance, those affecting the vestibulocerebellar system, those affecting the motor system (including tremor), cognitive impairment, and psychiatric and psychological adverse effects.^17^ All others were grouped as non‐CNS‐related TEAEs.

### 2.4 Risk of bias within studies and across studies

The Cochrane Risk of Bias 2 (RoB 2.0) tool was used to assess the quality of each RCT by two independent reviewers. Any discrepancy was resolved by a panel of adjudicators. The bias of each study was assessed either as “Low” or “High” or “Some concerns” based on the process of randomization followed, deviations from intended interventions, missing outcome data, measurement of the outcome, and selection of the reported result.^18^

The publication bias across the studies was evaluated using the funnel plots. For outcomes with ≥10 studies, the Egger's test was used to evaluate the publication bias, whereas visible asymmetry was applied for those with <10 studies.

### 2.5 Geometry of network

For each outcome, a network plot depicting the geometry of treatment networks across the trials was developed. Each circular node represents the type of treatment. The width of lines is proportional to the number of studies performing head‐to‐head comparisons in the same study.

### 2.6 Data synthesis and statistical analysis

All relative evidence was synthesized in a Bayesian framework, such as risk ratios (RRs) with 95% CIs for binary outcomes (efficacy, patient retention rate, and safety). NMA was performed using the “Gemtc” 4.0.4 package to run Bayesian analyses (R Foundation for Statistical Computing, Vienna, Austria). The clinical heterogeneity was assessed using the inconsistency index (*I* ^2^‐statistic) that describes the percentage of total variation across studies. Random effects model was used for the high level of heterogeneity (>50%) and fixed effects model was used for low and moderate (≤50%) levels of heterogeneity.

To rank the intervention, surface under the cumulative ranking curve (SUCRA) was calculated. SUCRA score of “one” is ranked as the best, whereas “zero” is ranked as the least. In terms of efficacy, the highest ranked ASM had greater efficacy than other ASMs and in terms of safety, the highest ranked ASM had the lowest risk compared with other ASMs. Sensitivity analyses for the 50% responder rate included analyses stratified by dose, median seizure frequency during baseline period, mean epilepsy duration, and mean age and were performed using meta v.4 software package.

## 3 RESULTS

### 3.1 Study selection

**FIGURE 1:**
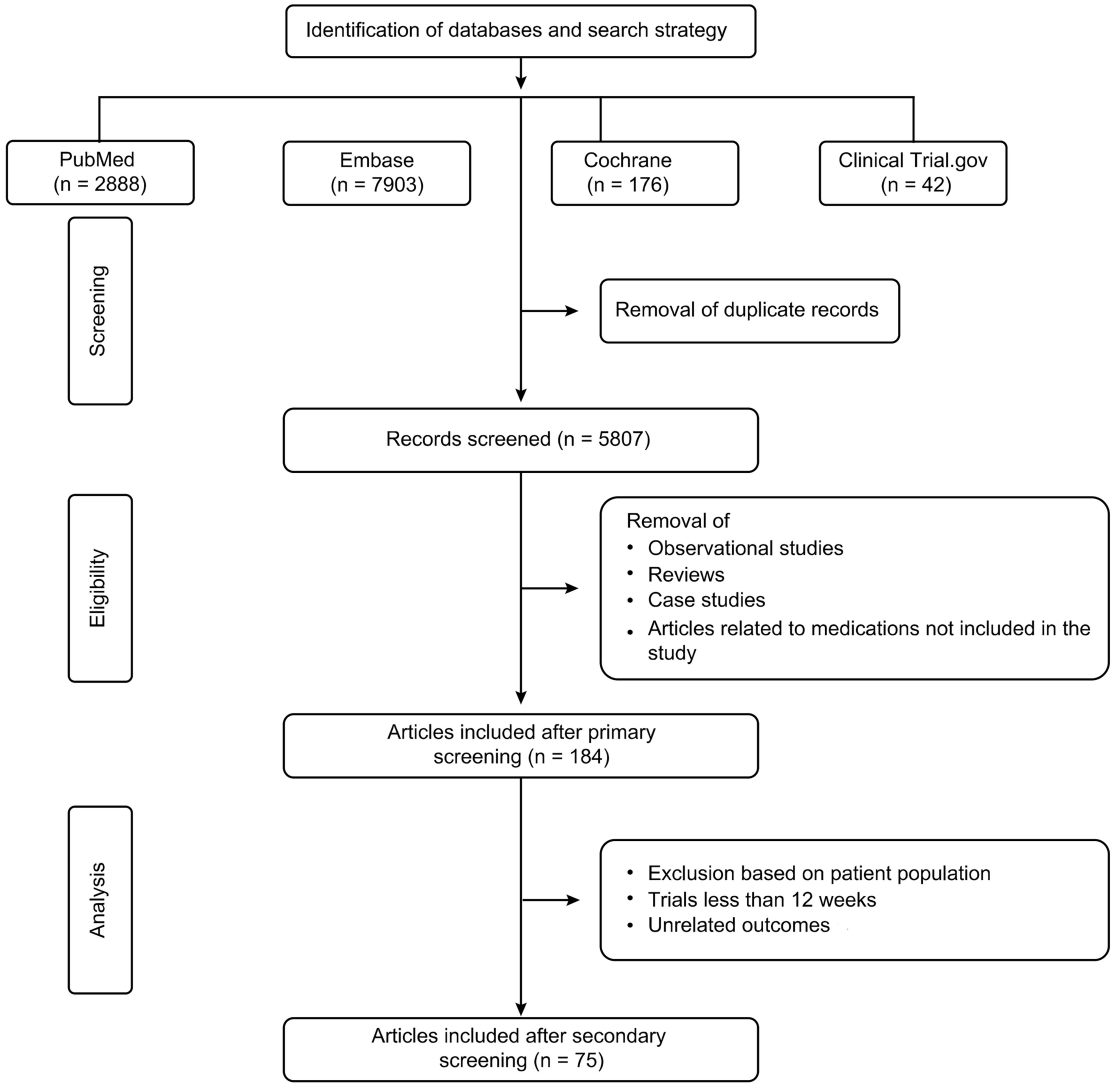
PRISMA flowchart.

The literature search yielded 5807 studies from the selected databases after removing duplicate articles. Articles were screened for relevancy based on the title and abstract that resulted in 184 studies. Full‐text screening of these articles resulted in 85 potentially relevant articles. Furthermore, based on the approved doses, 75 studies were considered eligible for the analysis. Figure 1 represents the study flow diagram as per the PRISMA guidelines.

### 3.2 Study characteristics

The baseline characteristics of the included articles were provided in Table S1. A total of 19 383 patients were included in the present analysis. There were four studies related to PER,^19^, ^20^, ^21^, ^22^ three related to RUF,^23^, ^24^, ^25^ five related to LTG,^26^, ^27^, ^28^, ^29^, ^30^ eight related to PGB,^31^, ^32^, ^33^, ^34^, ^35^, ^36^, ^37^, ^38^ four related to TGB,^39^, ^40^, ^41^, ^42^ five related to VGB,^43^, ^44^, ^45^, ^46^, ^47^ five related to GBP,^48^, ^49^, ^50^, ^51^, ^52^ six related to ZNS,^53^, ^54^, ^55^, ^56^, ^57^, ^58^ three related to BRV,^59^, ^60^, ^61^ four related to LCM,^62^, ^63^, ^64^, ^65^ eleven related to LEV,^66^, ^67^, ^68^, ^69^, ^70^, ^71^, ^72^, ^73^, ^74^, ^75^, ^76^ four related to EZG,^77^, ^78^, ^79^, ^80^ one related to CNB,^8^ two related to OXC,^81^, ^82^ five related to ESL,^83^, ^84^, ^85^, ^86^, ^87^ and five related to TPM,^88^, ^89^, ^90^, ^91^, ^92^ respectively. The age of the patients ranged from 12 to 80 years. Patient population consisted of either or all of the simple partial seizures, complex partial seizures, and secondary generalized seizures. The treatment period varied from 7 to 14 weeks.

### 3.3 Sensitivity analysis of baseline data

**FIGURE 2:**
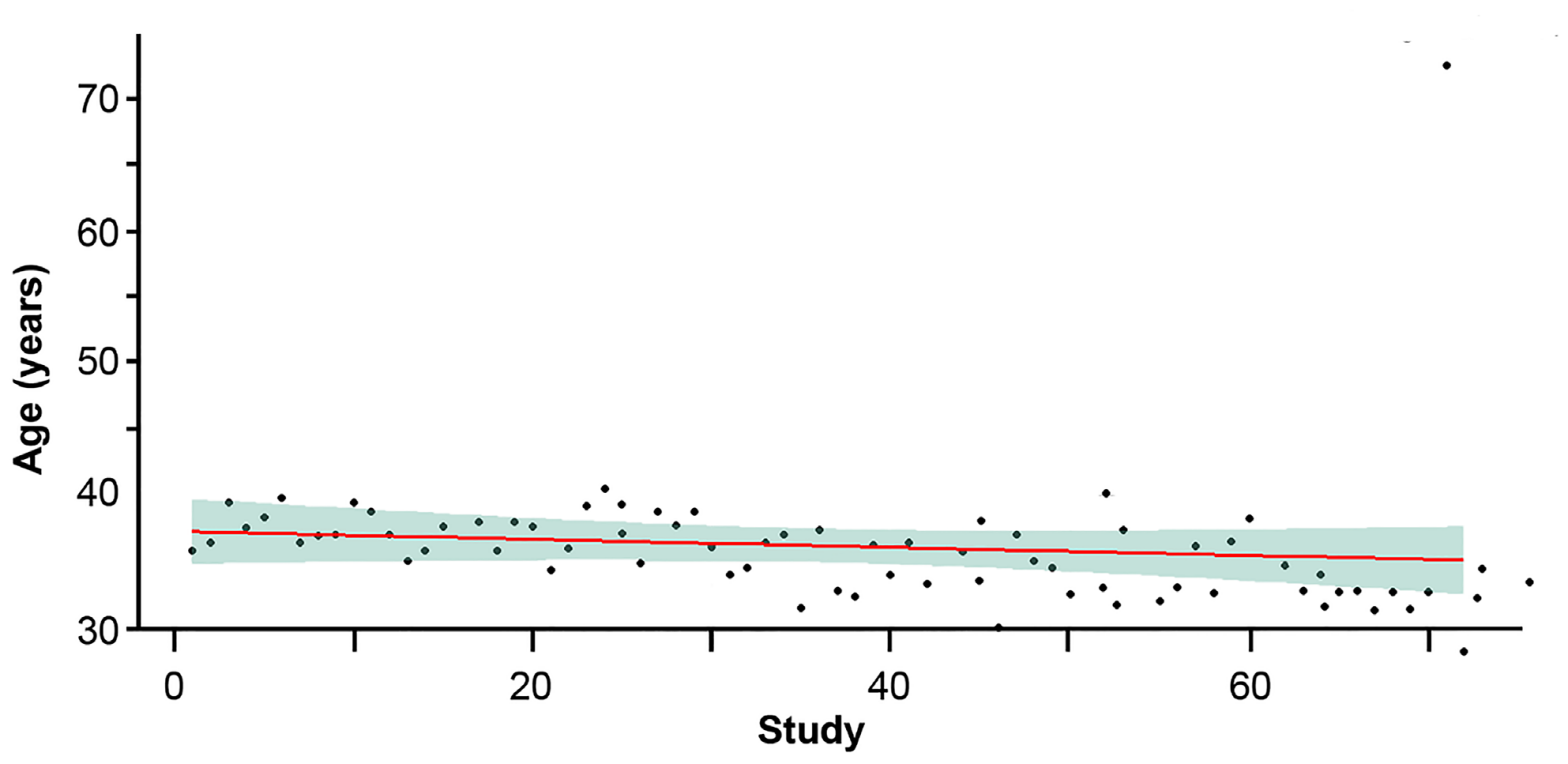
Sensitivity analysis based on mean age.

**FIGURE 3:**
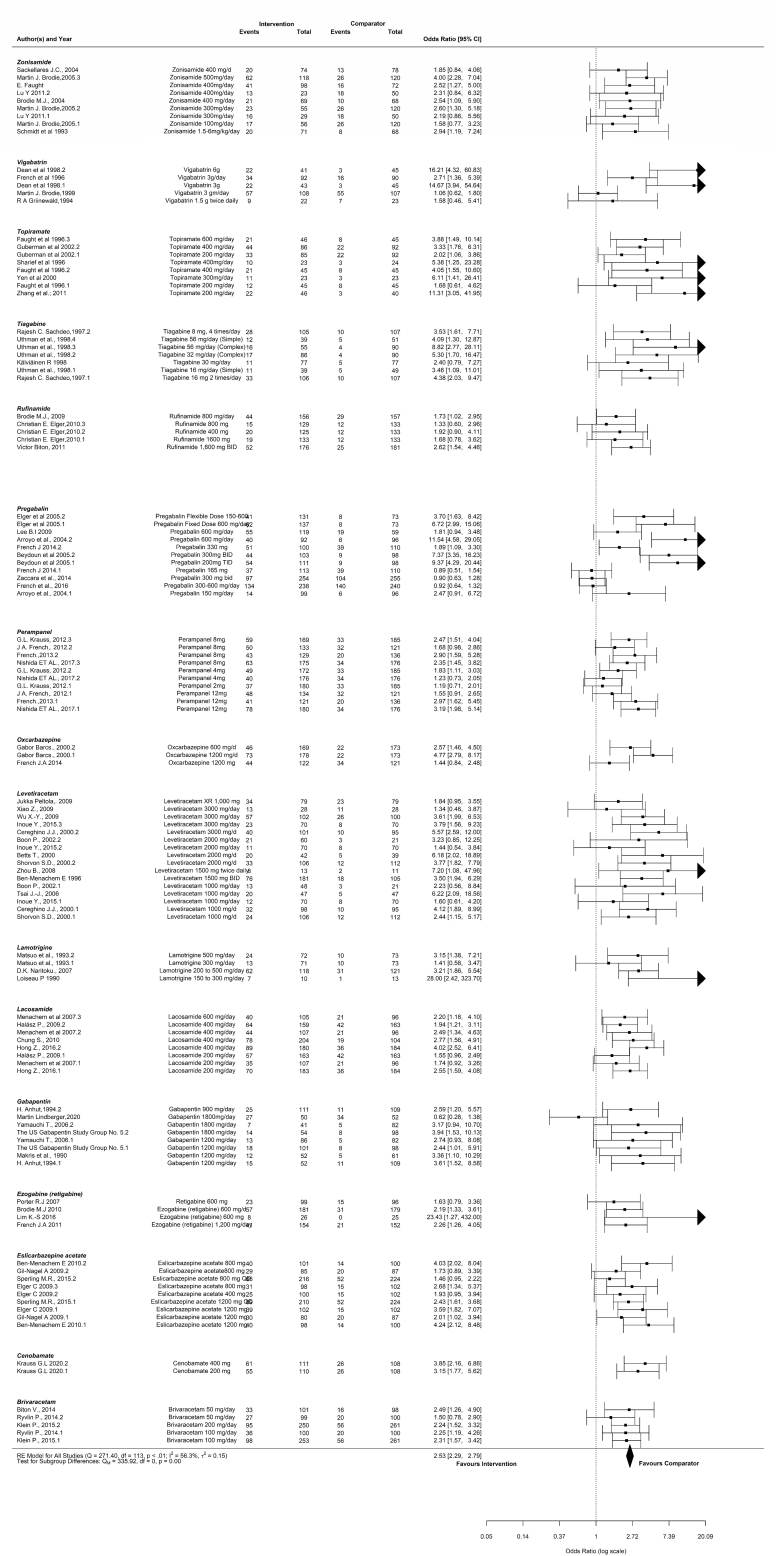
Sensitivity analysis based on dose.

Sensitivity analyses were conducted to examine the impact of heterogeneity across the studies included in the NMA. Sensitivity analyses stratified by the mean baseline age showed that the 50% responder rate did not vary significantly between the studies (Figure 2). Only the study conducted by Zhang et al. (2011)^93^ was an outlier, which included patients with a mean age of ~73 years, whereas in other studies the mean age was between 28 and 41 years. The duration of epilepsy and the mean seizure frequency during the baseline period were not reported in all studies; hence, sensitivity analysis was not performed based on these characteristics due to potential of selection bias. A dose‐dependent effect was observed for the 50% responder rate (Figure 3). However, studies conducted by Zaccara et al. (2014) (150–600 mg/day)^37^ and French et al. (2016) (150–600 mg/day)^36^ on PGB; Martin Lindberger et al. (2000) (1800 mg/day) on GBP^94^; and Elger et al. (2010) (200 mg/day) on RUF^24^ showed lesser effects than placebo, hence were considered as outliers in this analysis. The outliers in the sensitivity analysis were not considered for further efficacy and safety analysis. Therefore, a total of 75 articles were included in the final analysis.

### 3.4 Efficacy

**FIGURE 4:**
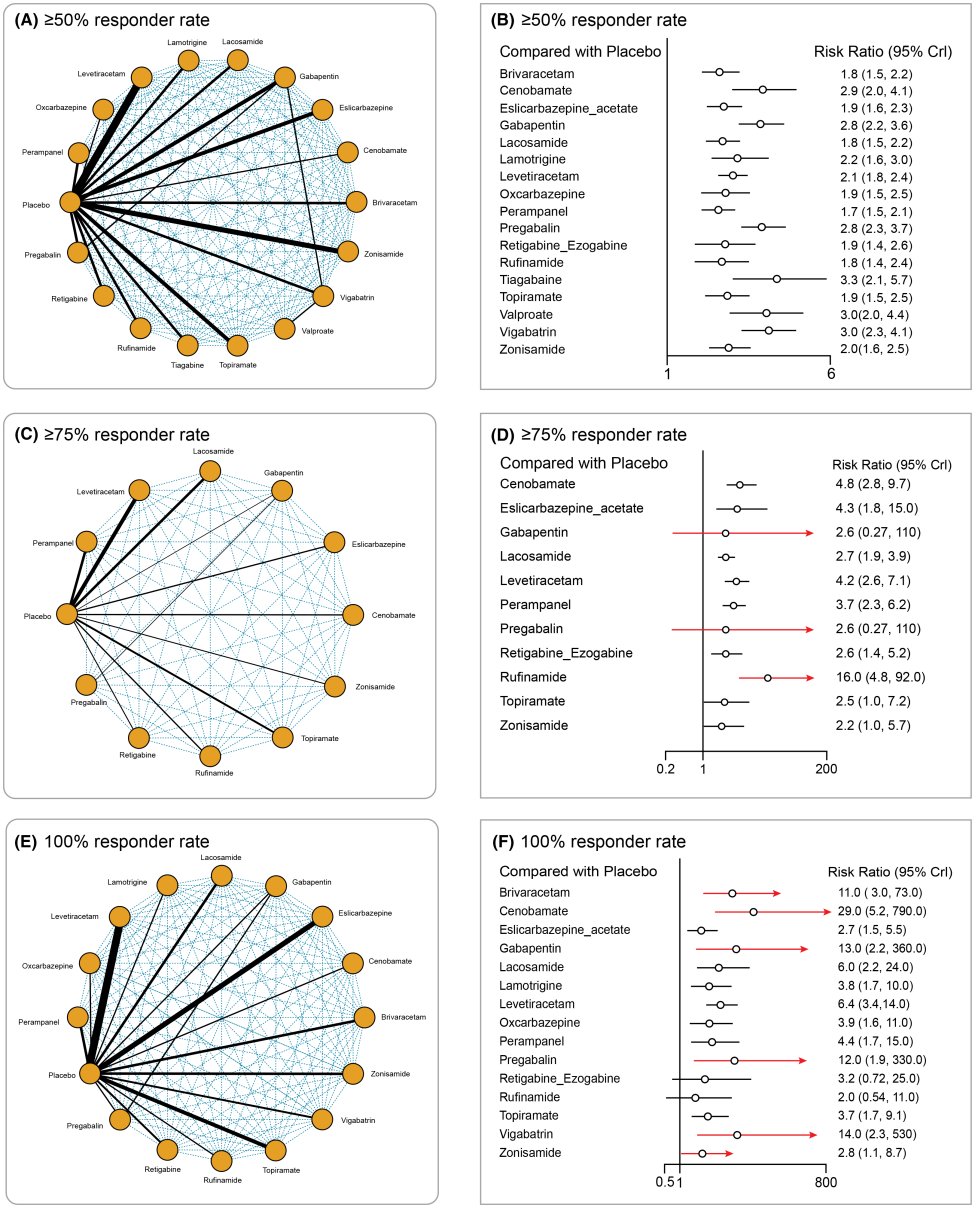
Multiple treatment comparison networks for efficacy outcomes. (A) ≥50% responder rate; (C) ≥75% responder rate; (E) 100% responder rate. Forest plots of efficacy outcomes. (B) ≥50% responder rate; (D) ≥75% responder rate; (F) 100% responder rate.

Overall, 71, 19, and 44 studies analyzed ≥50%, ≥75%, and 100% responder rates, respectively. Multiple treatment comparison networks are provided in Figure 4. Due to heterogeneity in outcome reporting between the included studies, 17 medication comparisons were available for ≥50% responder rate, 9 comparisons for ≥75% responder rate, and 15 comparisons for 100% responder rate. All interventions showed significantly higher ≥50% responder rate compared with placebo (Figure 4B). The RR (95% CI) was 1.80 (1.50–2.21) for BRV, 2.90 (2.04–4.13) for CNB, 1.90 (1.60–2.30) for ESL, 2.80 (2.21–3.60) for GBP, 1.84 (1.54–2.21) for LCM, 2.2 (1.64–3.04) for LTG, 2.1 (1.80–2.41) for LEV, 1.90 (1.50–2.50) for OXC, 1.74 (1.50–2.10) for PER, 2.82 (2.30–3.70) for PGB, 1.90 (1.40–2.63) for EZG, 1.82 (1.40–2.44) for RUF, 3.34 (2.10–5.73) for TGB, 1.93 (1.53–2.50) for TPM, 3.00 (2.00–4.44) for VPA, 3.03 (2.30–4.20) for VGB, and 2.00 (1.60–2.50) for ZNS compared with placebo. Among interventions, a significantly lower ≥50% responder rate was observed with BRV (Table S2) compared with PGB (0.63; 95% CI: 0.45–0.87) and TGB (0.53; 95% CI: 0.30–0.90); PER compared with GBP (0.63; 95% CI: 0.46–0.85), LEV (0.85; 95% CI: 0.67–6.11), PGB (0.62; 95% CI: 0.45–0.84), and TGB (0.52; 95% CI: 0.29–0.86).

Except ZNS, GBP, and PGB, all other interventions showed a significantly ≥75% higher responder rate compared with placebo (Figure 4D). For 100% responder rate, except for RGB and RUF, all other interventions were significant compared with placebo (Figure 4F). For ≥75% responder rate, the RR (95% CI) was 4.84 (2.80–9.70) for CNB, 4.34 (1.80–15.23) for ESL, 2.70 (1.90–3.90) for LCM, 4.20 (2.60–7.1) for LEV, 3.70 (2.34–6.21) for PER, 2.60 (1.4–5.20) for EZG, 16 (4.80–92.0) RUF, 2.52 (1.04–7.20) for TPM, and 2.23 (1.00–5.70) for ZNS compared with placebo. On comparing interventions, a significantly higher ≥75% responder rate was observed with RUF compared with LCM (6.02; 95% CI: 1.60–35.33), PER (4.24; 95% CI: 1.10–27.62), EZG (6.09; 95% CI: 1.44–38.90), TPM (6.24; 95% CI: 1.12–44.98), and ZNS (7.06; 95% CI: 1.51–48.18; Table S3). The 100% responder rate analysis showed RR (95% CI) of 11.00 (3.00–73.15) for BRV, 29.11 (5.20–794.9) for CNB, 2.7 (1.5–5.50) for ESL, 13.33 (2.20–356.2) for GBP, 6.00 (2.20–24.03) for LCM, 3.84 (1.71–10.40) for LTG, 6.44 (3.40–14.00) for LEV, 3.90 (1.60–11.00) for OXC, 4.40 (1.71–15.10) for PER, 12.16 (1.92–330.2) for PGB, 3.20 (0.72–25.47) for EZG, 2.03 (0.54–11.00) for RUF, 3.70 (1.75–9.13) for TPM, 14.18 (2.30–534.6) for VGB, and 2.80 (1.10–8.70) for ZNS. Among ASMs, CNB compared with ESL (10.71; 95% CI: 1.56–323.9) and ZNS (10.63; 95% CI: 1.37–261.2) showed a significantly higher 100% responder rate (Table S4). The fixed model effect was used for analyzing efficacy outcomes as the *I* ^2^ value was <50%. According to SUCRA, TGB, RUF, and CNB ranked the highest for ≥50%, ≥75%, and 100% responder rate, respectively, whereas placebo ranked the lowest for all three outcomes (Table S5).

### 3.5 Patient retention rate

**FIGURE 5:**
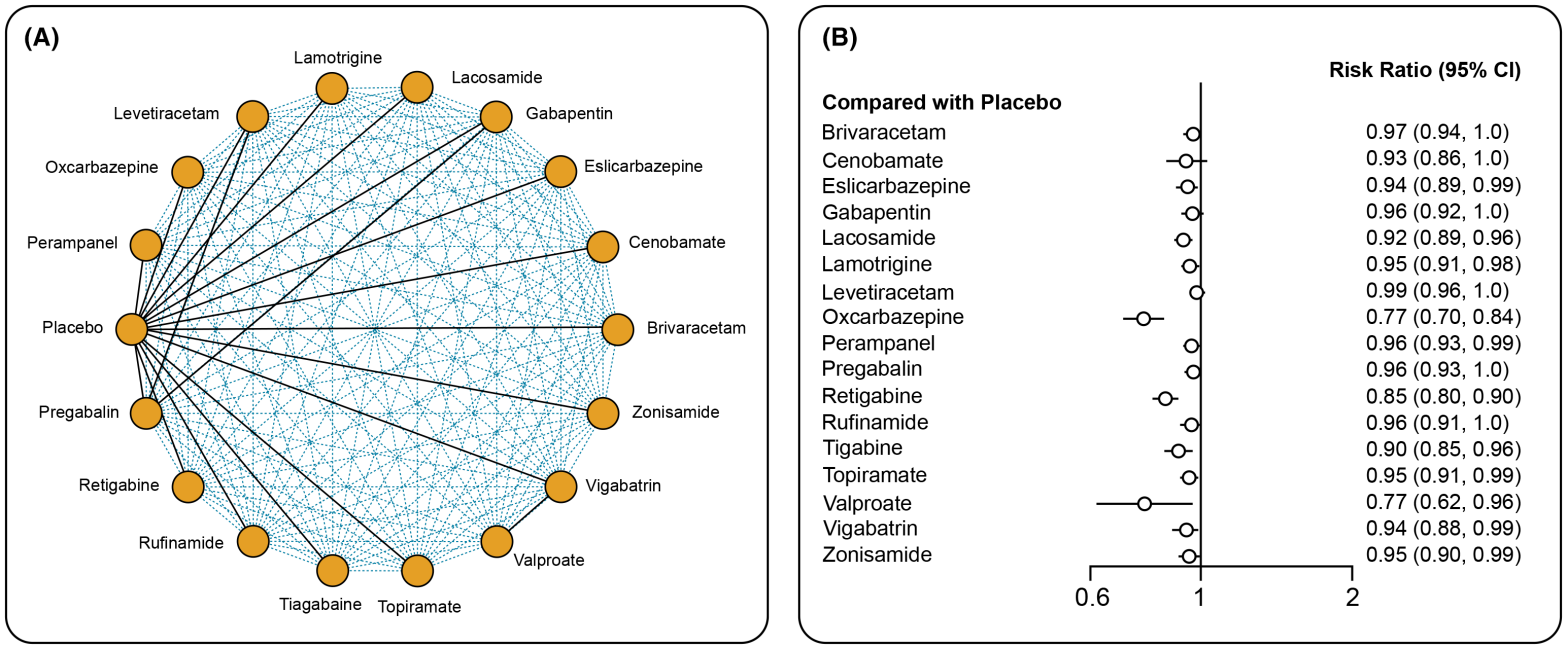
(A) Multiple treatment comparison networks for patient retention rate; (B) Forest plot for patient retention rate.

The network of interventions is represented in Figure 5A. Patient retention rate was evaluated from 71 studies. Four studies were excluded due to insufficient data.^45^, ^50^, ^57^, ^92^ Of all interventions, OXC, EZG, ESL, LCM, LTG, PER, TGB, TPM, VGB, and ZNS showed a statistically significant lower patient retention rate compared with placebo. The risk ratios for these interventions are shown in Figure 5B. The pair‐wise comparison between the treatments is represented in Table S6. OXC ranked the highest and placebo was the lowest as per SUCRA (Table S7). OXC had the least patient retention rate compared with other interventions (Table S6).

### 3.6 Safety

**FIGURE 6:**
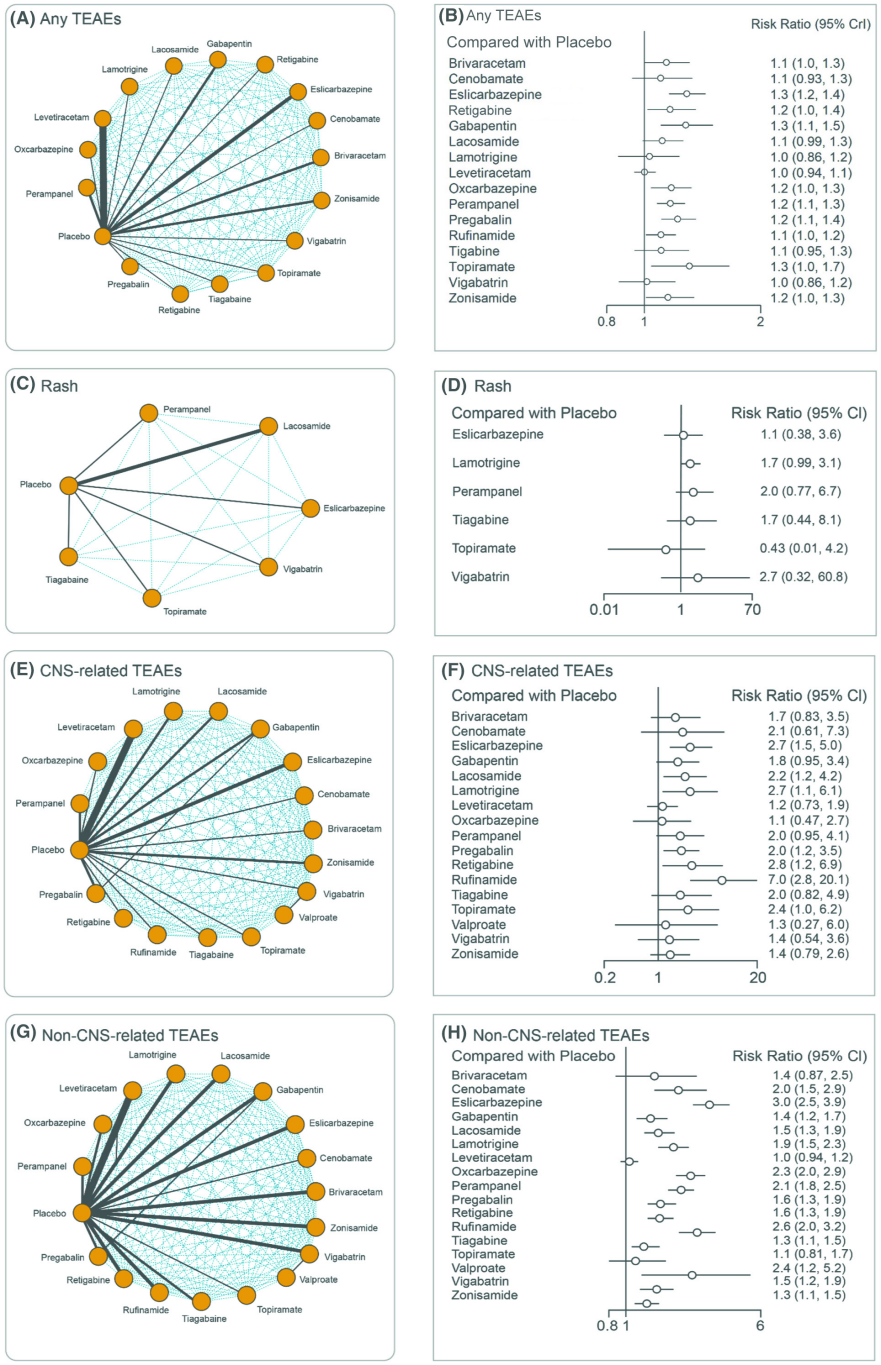
Multiple treatment comparison networks for safety outcomes. (A) Any TEAEs; (C) Rash; (E) CNS‐related TEAEs; (G) Non‐CNS‐related TEAEs. Forest plots of safety outcomes. (B) Any TEAEs; (D) Rash; (F) CNS‐related TEAEs; (H) Non‐CNS‐related TEAE.

The network of interventions for safety outcomes is represented in Figure 6A,C,E,G. For any TEAEs (50 studies), all the interventions safety were generally acceptable, LEV was ranked the highest (0.885067) after the placebo (Table S9) by SUCRA, indicating a lower risk compared with other ASMs.

For rash (12 studies), a numerically higher risk was observed with VGB (2.7; 95% CI: 0.32–60.84) and PER (2.0; 95% CI: 0.77–6.7) than other interventions compared with placebo. Although nonsignificant, TPM (0.43; 95% CI: 0.00–4.20) showed a lower risk of rash than others (Figure 6D), and the results were substantiated by SUCRA, ranking as the highest (0.830833; Table S9). The pair‐wise comparison of treatments for rash is presented in Table S10.

Compared with placebo, a significantly higher risk for CNS‐related TEAEs (55 studies) was observed with ESL (2.67; 95% CI: 1.46–5.01), LCM (2.26; 95% CI: 1.19–4.25), LTG (2.67; 95% CI: 1.46–5.01), PGB (2.04; 95% CI: 1.20–3.56), EZG (2.83; 95% CI: 1.13–7.02), and RUF (6.95; 95% CI: 2.75–20.13; Figure 6F). For non‐CNS‐related TEAEs (52 studies), when compared with placebo, all the interventions showed a significantly increased risk (Figure 6H) except for BRV (1.45; 95% CI: 0.87–2.53), LEV (1.05; 95% CI: 0.94–1.17), and TPM (1.14; 95% CI: 0.81–1.68). The pair‐wise comparison of treatments for CNS‐ and non‐CNS‐related TEAEs are presented in Tables S11 and S12, respectively. LEV was ranked the highest after placebo for both CNS‐ and non‐CNS‐related TEAEs, indicating the lowest risk compared with other ASMs (Table S9).

### 3.7 Risk of bias within studies and across studies

Study by Smith et al. (1993)^28^ showed a high risk of bias owing to the concerns over the randomization process and deviations from the intended intervention. Studies by Krauss et al. (2012)^21^ Loiseau et al. (1990)^26^ Matsuo et al. (1993)^29^ Sachdeo et al. (1997)^41^ Richens et al. (1995)^42^ showed a high risk of bias due to the process of randomization while Yen et al. (2000)^90^ owing to the deviations from the intended intervention. Overall, 50% of the studies were at low risk, 25% of the studies were with some concerns, and 25% of the studies were at high risk (Figures S1 and S2).

Publication bias across the studies was assessed by funnel plots obtained from the Egger's test. From the plots, publication bias was observed for efficacy outcomes (*p* > 0.05), whereas no publication bias was observed for safety outcomes (*p* < 0.05), except for non‐CNS‐related TEAEs (*p* = 0.08; Figures S3 and S4).

## 4 DISCUSSION

The present study provided the most comprehensive, up‐to‐date assessment of comparative efficacy and safety of all three generation ASMs approved as add‐on therapies for the management of focal epilepsy in adolescents and adult patients. The study included efficacy outcomes such as ≥50%, ≥75% and 100% responder rates; patient retention rate; and TEAE safety outcomes. The results of the NMA demonstrated that all ASMs were effective based on the 50% responder rate outcome. Further, an indirect comparison of different ASMs showed CNB as more efficacious in attaining seizure freedom. All ASMs showed a lower patient retention rate compared to placebo, with the least significant value observed for OXC. None of the ASMs, except BRV, showed statistically significant increased risk of incidence for overall TEAEs compared with placebo. To our knowledge, previous NMAs analyzed only a few endpoints or included limited interventions. This is the first study that has compared all three generations of approved ASMs to date, with extensive analysis of efficacy, safety and retention rate outcomes.

Responder rate of ≥50% reduction in seizure frequency is commonly used for evaluation of efficacy of ASMs. Our results showed all approved ASMs were effective as add‐on treatment for focal epilepsy, with significantly higher ≥50% responder rate compared with placebo, which is in line with previous publications.^2^, ^12^, ^14^, ^15^ The current study showed that TGB had the highest ≥50% reduction in seizure frequency compared with placebo, which is in agreement with published meta‐analysis.^19^ Nevertheless, the studies on TGB considered for the present NMA showed a high risk of bias. More studies are needed to demonstrate their effectiveness.

Interestingly, except CNB, most newer ASMs didn't show better efficacy of ≥50% responder rate than older ASMs. Efficacy evaluation of the newer generation ASMs is usually performed by adding the new ASM or placebo to the baseline medications that can mostly be the second‐generation drugs in patients with refractory epilepsy; responder rate is compared between the active treatment and pretreatment baseline periods between the ASMs‐ and placebo‐treated groups.^95^ Previous studies have reported a decrease in efficacy with each additional ASM added to an existing ASM regimen.^96^, ^97^ However, the CNB study included in this analysis also included patients receiving second‐generation drugs such as VPA, LTG, CBZ and OXC.^8^ The same was observed in other studies with other third‐generation drugs where patients had previously received the second‐generation ASMs with improved efficacy.^19^, ^22^, ^59^, ^63^, ^84^

The ultimate treatment goal of ASMs is to attain seizure freedom. Compared with placebo, CNB showed the highest odds of achieving seizure freedom, which is in accordance with the published literature. Studies have reported a better seizure freedom with CNB during the maintenance phase (CNB 400 mg/day vs Placebo: 21% vs 1%, *p* < 0.0001) and also a sustained effect during the extension phase of 4 years.^8^, ^98^ A recent NMA comparing the third‐generation ASMs showed that CNB is the best ASM in terms of efficacy outcomes.^12^ However, only third‐generation ASMs were investigated in previous study, whereas our current NMA showed CNB performed best efficacy in seizure freedom across all three generation AMSs. High seizure freedom response of CNB was further corroborated in a recent systematic review based on the real‐world studies.^99^ The better seizure freedom observed with CNB compared with other ASMs may be attributed to its unique dual complementary mechanism of action on targets that regulate both excitatory neurotransmission and inhibitory neurotransmission.^95^

The current study also analyzed outcome of patient retention rate, which reflects both drug effectiveness and tolerability.^100^ Overall, the second‐ and third‐generation ASMs showed better patient retention toward the end of the study compared with placebo, suggesting higher acceptability of newer ASMs. So far, there is not much reporting on the comparative patient retention rate of approved ASMs. Patient retention rate of PER and CBZ on focal epilepsy has been reported^100^, ^101^; however, both studies have reported the retention rate from studies only covering one specific intervention (PER and CBZ). Hence, the present study provides a valuable insight of comparative patient retention rate for multiple interventions in adult patients with epilepsy in a double‐blind setting.

Regarding safety, all drugs showed the acceptable tolerability, placebo and LEV have the lowest risk which is consistent with previous studies.^2^, ^102^ As TEAE of interest, risk of rash was specifically analyzed. Interestingly, we observed that VGB and PER showed a numerically higher risk of rash compared with sodium channel blockers, such as LTG and ESL, which is inconsistent with previous report that sodium channel blockers were associated with higher risk of rash.^103^ This discrepancy was possibly due to the number of studies included, relatively small sample size, and lack of long‐term data. In fact, rash was reported in only few studies; hence, most of the ASMs could not be evaluated.

To maintain consistency and homogeneity, trials with relatively similar study design and clinical characteristics were included in the analysis. As data of different doses in the same study were pooled and considered as a single value, sensitivity analysis stratified by dose as well as age was performed to analyze their influence on the efficacy, and the outliers were excluded from the study for further analysis to maintain consistency. Nonetheless, cautions still should be made considering the present study included different RCTs spanning nearly 30 years.

The study has certain limitations. First, most of RCTs included in the present study were placebo‐controlled instead of active‐controlled. Indirect comparisons in this study may impede the consistency assumption with few exceptions. In case of CNB, only one RCT was eligible for analysis. Hence, the comparison between treatment should be interpreted with caution. Clinical decisions should be based on comprehensive consideration of RCTs, NMAs and real‐world evidence. Besides, the present study didn't conduct analysis in pediatric population and didn't consider QoL as an outcome. Further studies should be conducted in the future. Moreover, the search was limited to English‐based articles.

## 5 CONCLUSION

All approved ASMs were effective to varying degrees compared with placebo as add‐on therapy in focal epilepsy. Among the included ASMs, CNB had the greatest likelihood of allowing patients to attain seizure freedom.

## AUTHOR CONTRIBUTIONS

All authors made a significant contribution to the work reported, whether that is in the conception, study design, execution, acquisition of data, analysis and interpretation, or in all these areas; took part in drafting, revising, or critically reviewing the article; gave final approval of the version to be published; have agreed on the journal to which the article has been submitted; and agreed to be accountable for all aspects of the work.

## FUNDING INFORMATION

This study was funded by National Natural Science Foundation of China (Grant number: U21A20393) and Science & Technology Department of Sichuan Province (Grant number: 2023YFQ0109). Statistical analysis and medical writing assistance were funded by Ignis Therapeutics.

## CONFLICT OF INTEREST STATEMENT

All authors have read and approved the manuscript. Yutong Liu and Hui Ye are employees of Ignis Therapeutics (Shanghai) Limited, China. The other authors report no other potential conflicts of interest in this work. We confirm that we have read the Journal's position on issues involved in ethical publication and affirm that this report is consistent with those guidelines.

## ETHICS STATEMENT

Not applicable.

## PATIENT CONSENT STATEMENT

Not applicable.

## PERMISSION TO REPRODUCE MATERIAL FROM OTHER SOURCES

Not applicable.

## Supporting information


Data S1.

## Data Availability

The data that support the findings of this study are available from the corresponding author upon reasonable request.
