# Supplementary material for: Efficacy and safety of add‐on antiseizure medications for focal epilepsy: A network meta‐analysis
Source: Epilepsia Open. 2024 Jun 18;9(4):1550–64. doi: 10.1002/epi4.12997 (PMC11296132; doi:10.1002/epi4.12997)
Supplement: Supplementary file 1 — Data S1. [file EPI4-9-1550-s001.docx]

**Supplementary methods**

**Search string:**

An extensive literature search was performed in different databases like PubMed, Embase, Cochrane and clinicaltrial.gov for articles published till March 2023 using the following **PUBMED:**

((((((((((((((((((((((((((((((((focal epilepsy[MeSH Terms]) OR (refractory focal epilepsy[MeSH Terms])) OR (drug-resistant focal epilepsy[MeSH Terms])) OR (partial epilepsy[MeSH Terms])) AND (Pregabalin[MeSH Terms])) OR (Tiagabine[MeSH Terms])) OR (vigabatrin[MeSH Terms])) OR (zonisamide[MeSH Terms])) OR (lacosamide[MeSH Terms])) OR (lyrica[MeSH Terms])) OR (tiagabine[MeSH Terms])) OR (gabitril[MeSH Terms])) OR (sabril[MeSH Terms])) OR (zonegran[MeSH Terms])) OR (vimpat[MeSH Terms])) OR (brivaracetam[MeSH Terms])) OR (briviact[MeSH Terms])) OR (cenobamate[MeSH Terms])) OR (xcopri[MeSH Terms])) OR (eslicarbazepine[MeSH Terms])) OR (aptiom[MeSH Terms])) OR (Levetiracetam[MeSH Terms])) OR (keppra[MeSH Terms])) OR (Oxcarbazepine[MeSH Terms])) OR (Topiramate[MeSH Terms])) OR (Lamotrigine[MeSH Terms])) OR (carbamazepine[MeSH Terms])) OR (valproate[MeSH Terms])) OR (rufinamide[MeSH Terms])) OR (gabapentin[MeSH Terms])) OR (perampanel[MeSH Terms])) OR (retigabine[MeSH Terms])) OR (carisbamate[MeSH Terms])

Filters applied: Randomized Controlled Trial

**EMBASE:**

(('focal epilepsy'/exp OR 'epilepsies, partial' OR 'epilepsy, partial' OR 'focal epilepsy' OR 'local epilepsy' OR 'localization-related epilepsies' OR 'localization-related epilepsy' OR 'partial epilepsies' OR 'partial epilepsy') OR ('focal seizures'/exp OR 'focal fits' OR 'focal onset seizure' OR 'focal seizure' OR 'focal seizures' OR 'local seizure' OR 'localized seizure' OR 'partial seizure' OR 'seizure, partial') OR (adult/exp OR 'adult' OR 'adults' OR 'grown-ups' OR 'grownup' OR 'grownups') OR ('adjuvant therapy'/exp OR 'adjuvant effect' OR 'adjuvant therapy' OR 'adjuvant treatment') OR ('refractory focal epilepsy'/exp OR 'drug resistant focal epilepsies' OR 'drug resistant focal epilepsy' OR 'drug resistant partial epilepsies' OR 'drug resistant partial epilepsy' OR 'intractable focal epilepsies' OR 'intractable focal epilepsy' OR 'intractable partial epilepsies' OR 'intractable partial epilepsy' OR 'pharmacoresistent focal epilepsy' OR 'pharmacoresistent partial epilepsy' OR 'refractory focal epilepsies' OR 'refractory focal epilepsy' OR 'refractory partial epilepsies' OR 'refractory partial epilepsy' OR 'resistant focal epilepsies' OR 'resistant focal epilepsy' OR 'resistant partial epilepsies' OR 'resistant partial epilepsy')) AND ((pregabalin/exp OR '3 aminomethyl 5 methylhexanoic acid' OR '3 isobutyl 4 aminobutyric acid' OR '3 isobutyl GABA' OR '3 isobutylgaba' OR '4 amino 3 isobutylbutyric acid' OR 'bonqat' OR 'ci 1008' OR 'ci1008' OR 'lyrica' OR 'lyrica cr' OR 'pd 144723' OR 'pd144723' OR 'pregabalin' OR 'vronogabic' OR 'ynp 1807' OR 'ynp1807') OR (tiagabine/exp OR '1 [4, 4 bis (3 methyl 2 thienyl) 3 butenyl] 3 piperidinecarboxylic acid' OR '1 [4, 4 bis (3 methyl 2 thienyl) 3 butenyl] nipecotic acid' OR 'cep 6671' OR 'cep6671' OR 'gabatril' OR 'gabitril' OR 'n [4, 4 bis (3 methylthien 2 yl) but 3 enyl] nipecotic acid' OR 'nnc 05 0328' OR 'nnc 328' OR 'no 05 0328' OR 'no 05 0329' OR 'no 050328' OR 'no 328' OR 'no 329' OR 'no050328' OR 'tiabex' OR 'tiagabine' OR 'tiagabine hydrochloride') OR (zonisamide/exp OR '1, 2 benzisoxazole 3 methanesulfonamide' OR '1, 2 benzisoxazole, 3 sulfamoylmethyl' OR '3 sulfamoylmethyl 1, 2 benzisoxazole' OR 'ad 810' OR 'ad810' OR 'benzo [d] isoxazol 3 ylmethanesulfonamide' OR 'ci 912' OR 'ci912' OR 'cinal' OR 'desizon' OR 'ersittin' OR 'et 104' OR 'et104' OR 'excegran' OR 'excemid' OR 'kinaplase' OR 'nyzol' OR 'pd 110843' OR 'pd110843' OR 'tremode' OR 'trerief' OR 'zonegran' OR 'zonesme' OR 'zonibon' OR 'zonisade' OR 'zonisahexal' OR 'zonisamide' OR 'zonisol') OR (lacosamide/exp OR '2 (acetamido) 3 methoxy n (phenylmethyl) propanamide' OR '2 (acetylamino) 3 methoxy n (phenylmethyl) propanamide' OR '2 acetamido n benzyl 3 methoxypropionamide' OR '2 acetylamino n benzyl 3 methoxypropanamide' OR 'add 234037' OR 'add234037' OR 'ads 4101' OR 'ads4101' OR 'arkvimma' OR 'cosim' OR 'eplaid' OR 'erlosamide' OR 'harkoseride' OR 'kanilad' OR 'lackepila' OR 'lacoala' OR 'lacopat' OR 'lacosabil' OR 'lacosadel' OR 'lacosamide' OR 'lacosamide pain' OR 'laprysta' OR 'lendenuz' OR 'losmorid' OR 'lydraso' OR 'midza' OR 'n acetyl o methyl dextro serine benzylamide' OR 'seizpat' OR 'spm 927' OR 'spm 929' OR 'spm927' OR 'spm929' OR 'stutan' OR 'trelema' OR 'vilepsia' OR 'vimpat' OR 'vimpato' OR 'zilibra') OR (brivaracetam/exp OR '2 (2 oxo 4 propyl 1 pyrrolidinyl) butanamide' OR '2 [2 oxo 4 propylpyrrolidin 1 yl] butanamide' OR '2 [2 oxo 4 propyltetrahydro 1h pyrrol 1 yl] butanamide' OR 'alpha ethyl 2 oxo 4 propyl 1 pyrrolidineacetamide' OR 'brivaracetam' OR 'briviact' OR 'brivlera' OR 'nubriveo' OR 'rikelta' OR 'ucb 34714' OR 'ucb34714') OR (cenobamate/exp OR '1 (2 chlorophenyl) 2 (2h tetrazol 2 yl) ethyl carbamate' OR '[1 (2 chlorophenyl) 2 (tetrazol 2 yl) ethyl] carbamate' OR 'alpha (2 chlorophenyl) 2h tetrazole 2 ethanol 2 carbamate' OR 'carbamic acid 1 (2 chlorophenyl) 2 (2h tetrazol 2 yl) ethyl ester' OR 'cenobamate' OR 'ono 2017' OR 'ono2017' OR 'ontozry' OR 'xcopri' OR 'ykp 3089' OR 'ykp3089') OR ('eslicarbazepine acetate'/exp OR '10 acetoxy 10, 11 dihydro 5h dibenz [b, f] azepine 5 carboxamide' OR '5 (aminocarbonyl) 10, 11 dihydro 5h dibenzo [b, f] azepin 10 yl acetate' OR '[11 carbamoyl 5, 6 dihydrobenzo [b] [1] benzazepin 5 yl] acetate' OR 'acetic acid 5 carbamoyl 10, 11 dihydro 5h dibenzo [b, f] azepin 10 yl ester' OR 'aptiom' OR 'bia 2 093' OR 'bia 2-093' OR 'bia 2093' OR 'bia2 093' OR 'bia2-093' OR 'bia2093' OR 'eslicarbazepine acetate' OR 'exalief' OR 'piolex' OR 'sep 0002093' OR 'sep0002093' OR 'stedesa' OR 'zebinix') OR (levetiracetam/exp OR 'agb 101' OR 'agb101' OR 'desitrend' OR 'e keppra' OR 'elepsia' OR 'elepsia xr' OR 'keppra' OR 'keppra xr' OR 'kopodex' OR 'l 059' OR 'l059' OR 'levesam' OR 'levetiracetam' OR 'levetiracetam in sodium chloride' OR 'levipil' OR 'levroxa' OR 'lo 59' OR 'lo59' OR 'matever' OR 'spritam' OR 'ucb 059' OR 'ucb 22059' OR 'ucb l 059' OR 'ucb l059' OR 'ucb059' OR 'ucb22059') OR (oxcarbazepine/exp OR '10, 11 dihydro 10 oxo 5h dibenz [b, f] azepine 5 carboxamide' OR '10, 11 dihydro 10 oxocarbamazepine' OR '5 carbamoyl 10, 11 dihydro 10 oxo 5h dibenz [b, f] azepine' OR '5 oxo 6h benzo [b] [1] benzazepine 11 carboxamide' OR 'apydan' OR 'carbamazepine, 10, 11 dihydro 10 oxo' OR 'epliga' OR 'gp 47680' OR 'gp47680' OR 'karbagen' OR 'kin 493' OR 'kin493' OR 'npc 04' OR 'npc04' OR 'oxalept' OR 'oxcarbazepine' OR 'oxepilax' OR 'oxocarbazepine' OR 'oxrate' OR 'oxtellar' OR 'oxtellar xr' OR 'spn 604' OR 'spn 804' OR 'spn604' OR 'spn804' OR 'timox' OR 'tolep' OR 'tri 476' OR 'tri476' OR 'trileptal' OR 'trileptin' OR 'zigabal') OR (topiramate/exp OR '(4, 4, 11, 11 tetramethyl 3, 5, 7, 10, 12 pentaoxatricyclo [7.3.0.0 (2, 6)] dodecan 6 yl) methyl sulfamate' OR '2, 3:4, 5 bis o (1 methylethylidene) beta dextro fructopyranose sulfamate' OR '2, 3:4, 5 di o isopropylidene beta dextro fructopyranose sulfamate' OR 'acomicil' OR 'ecuram' OR 'epiramat' OR 'epitomax' OR 'epitoram' OR 'eprontia' OR 'erravia' OR 'et 101' OR 'et101' OR 'etopro' OR 'fagodol' OR 'jadix' OR 'kw 6485' OR 'kw6485' OR 'lusitrax' OR 'maritop' OR 'mcn 4853' OR 'mcn4853' OR 'oritop' OR 'piraleps' OR 'pirantal' OR 'pirepil' OR 'qudexy' OR 'qudexy xr' OR 'ramas (drug)' OR 'rwj 17021' OR 'rwj 17021-000' OR 'rwj17021' OR 'rwj17021-000' OR 'sincronil' OR 'spn 538' OR 'spn538' OR 'talopam' OR 'tiramat' OR 'topaben' OR 'topamac' OR 'topamax' OR 'topamax sprinkle' OR 'topepsil' OR 'topibrain' OR 'topilek' OR 'topimark' OR 'topimax' OR 'topina' OR 'topiramat' OR 'topiramat-ct' OR 'topiramate' OR 'topiramato' OR 'topiratore' OR 'topit' OR 'toramat' OR 'torlepta' OR 'trokendi' OR 'trokendi xr' OR 'trokesa' OR 'usl 255' OR 'usl255') OR (lamotrigine/exp OR '3, 5 diamino 6 (2, 3 dichlorophenyl) 1, 2, 4 triazine' OR '6 (2, 3 dichlorophenyl) 1, 2, 4 triazine 3, 5 diamine' OR 'arvind' OR 'auc 025' OR 'auc025' OR 'bw 430 c' OR 'bw 430c' OR 'bw 430c78' OR 'bw430c' OR 'bw430c78' OR 'crisomet' OR 'dezepil' OR 'et 105' OR 'et105' OR 'eur 1048' OR 'eur1048' OR 'gerolamic' OR 'gw 273293' OR 'gw273293' OR 'labileno' OR 'lamal' OR 'lambipol' OR 'lamepil' OR 'lamictal' OR 'lamictal cd' OR 'lamictal odt' OR 'lamictal xr' OR 'lamictin' OR 'lamilept' OR 'lamitrin' OR 'lamitrin s' OR 'lamodex' OR 'lamogine' OR 'lamolep' OR 'lamosynt' OR 'lamotrigin' OR 'lamotrigine' OR 'lamotrin' OR 'lamotrix' OR 'larig' OR 'medotrigin' OR 'neurium' OR 'plexxo' OR 'seizal (drug)' OR 'subvenite' OR 'symla') OR (carbamazepine/exp OR '(5h) dibenz (b, f) azepine 5 carboxamide' OR '5 carbamoyl 5h dibenz [b, f] azepine' OR '5h dibenz [b, f] azepine 5 carboxamide' OR 'amizepin' OR 'amizepine' OR 'apo-carbamazepine' OR 'atretol' OR 'bipotrol' OR 'biston' OR 'calepsin' OR 'camapine' OR 'carbadac' OR 'carbadura' OR 'carbagen' OR 'carbalex' OR 'carbamazepin' OR 'carbamazepine' OR 'carbategral' OR 'carbatol' OR 'carbatrol' OR 'carbazene' OR 'carbazep' OR 'carbazina' OR 'carbella' OR 'carbepsil' OR 'carmaz' OR 'carnexiv' OR 'carpaz' OR 'carzepin' OR 'carzepine' OR 'clostedal' OR 'convuline (carbamazepine)' OR 'curatil' OR 'epileptol' OR 'epileptol cr' OR 'epimax' OR 'epitol' OR 'eposal retard' OR 'equetro' OR 'espa-lepsin' OR 'finlepsin' OR 'foxalepsin' OR 'foxalepsin retard' OR 'g 32883' OR 'g32883' OR 'hermolepsin' OR 'karbamazepin' OR 'karbelex' OR 'kodapan' OR 'lexin' OR 'mazepine' OR 'mazetol' OR 'neugeron' OR 'neurotol' OR 'neurotop' OR 'neurotop retard' OR 'nordotol' OR 'painbrake (drug)' OR 'panitol' OR 'servimazepin' OR 'sirtal' OR 'spd 417' OR 'spd417' OR 'stazepine' OR 'storilat' OR 'syntopine' OR 'tardotol' OR 'taver' OR 'tegol' OR 'tegral' OR 'tegretal' OR 'tegretol' OR 'tegretol cr' OR 'tegretol divitab' OR 'tegretol retard' OR 'tegretol xr' OR 'tegretol-s' OR 'tegretol-xr' OR 'tegrital' OR 'telesmin' OR 'temporal slow' OR 'temporol' OR 'teril' OR 'timonil' OR 'timonil retard') OR ('valproic acid'/exp OR '2 propylpentanoate' OR '2 propylpentanoic acid' OR '2 propylvalerate sodium' OR '2 propylvaleric acid' OR '2 propylvaleric acid sodium' OR '2, 2 dipropyl acetic acid' OR '4 heptanecarboxylic acid' OR 'abbott 44090' OR 'absenor' OR 'absenor depot' OR 'alpha propylvalerate' OR 'alpha propylvaleric acid' OR 'apilepsin' OR 'atemperator' OR 'convival chrono' OR 'convulex' OR 'convulex chrono' OR 'convulex cr' OR 'convulex retard' OR 'cs 1' OR 'cs1' OR 'ct 010' OR 'ct010' OR 'delepsine' OR 'delepsine retard' OR 'depacon' OR 'depakene' OR 'depakene chrono' OR 'depakin' OR 'depakin chrono' OR 'depakine' OR 'depakine chrono' OR 'depakine chrono retard' OR 'depakine chronosphere' OR 'depakine crono' OR 'depakine druppels' OR 'depakine enteric' OR 'depakine iv' OR 'depalept' OR 'depalept chrono' OR 'deprakine' OR 'deprakine retard' OR 'di n propylacetate' OR 'di n propylacetate sodium' OR 'di n propylacetic acid' OR 'diplexil' OR 'dipropyl acetic acid' OR 'dipropylacetate' OR 'dipropylacetate sodium' OR 'dipropylacetatic acid' OR 'dipropylacetic acid' OR 'diprosin' OR 'dyzantil' OR 'epilam' OR 'epilex' OR 'epilim' OR 'epilim chrono' OR 'epilim chrono 500' OR 'epilim chronosphere' OR 'epilim chronosphere mr' OR 'epilim enteric' OR 'episenta' OR 'epival cr' OR 'ergenyl' OR 'ergenyl chrono' OR 'ergenyl chronosphere' OR 'ergenyl retard' OR 'espa valept' OR 'espa-valept' OR 'everiden' OR 'goilim' OR 'hexaquin' OR 'k 828' OR 'k 828ab' OR 'k 828sp' OR 'k828' OR 'k828ab' OR 'k828sp' OR 'kw 6066 n' OR 'labazene' OR 'leptilan' OR 'leptilanil' OR 'micropakine' OR 'micropakine lp' OR 'mylproin' OR 'myproic acid' OR 'n dipropylacetic acid' OR 'npl 2005' OR 'npl2005' OR 'orfil' OR 'orfiril' OR 'orfiril cr' OR 'orfiril iv' OR 'orfiril long' OR 'orfiril retard' OR 'orlept' OR 'petilin' OR 'propymal' OR 'propymal enteric' OR 'sodium 2 propylpentanoate' OR 'sodium 2 propylvalerate' OR 'sodium di n propyl acetate' OR 'sodium di n propylacetate' OR 'sodium dipropyl acetate' OR 'sodium dipropylacetate' OR 'sodium n dipropylacetate' OR 'sodium valproate' OR 'sodium valproate plus valproic acid' OR 'sodium valproate/valproic acid' OR 'stavzor' OR 'syonell' OR 'val 001 (anticonvulsive agent)' OR 'val001 (anticonvulsive agent)' OR 'valberg pr' OR 'valcote' OR 'valepil' OR 'valeptol' OR 'valerin' OR 'valhel pr' OR 'valoin' OR 'valpakine' OR 'valparin' OR 'valporal' OR 'valprax' OR 'valpro' OR 'valpro al' OR 'valpro beta' OR 'valproat' OR 'valproat chrono' OR 'valproate' OR 'valproate retard' OR 'valproate sodium' OR 'valproate sodium plus valproic acid' OR 'valproate sodium/valproic acid' OR 'valprodura' OR 'valproic acid' OR 'valproic acid plus sodium valproate' OR 'valproic acid plus valproate sodium' OR 'valproic acid/sodium valproate' OR 'valproic acid/valproate sodium' OR 'valprosid' OR 'valprotek' OR 'valprotek cr' OR 'valsup' OR 'vupral') OR (rufinamide/exp OR '1 (2, 6 difluorobenzyl) 1, 2, 3 triazole 4 carboxamide' OR '1 (2, 6 difluorobenzyl) 1h 1, 2, 3 triazole 4 carboxamide' OR '1 [ (2, 6 difluorophenyl) methyl] triazole 4 carboxamide' OR 'banzel' OR 'cgp 33101' OR 'cgp33101' OR 'e 2080' OR 'e2080' OR 'inovelon' OR 'ruf 331' OR 'ruf331' OR 'rufinamide' OR 'syn 111' OR 'syn111' OR 'xilep') OR (gabapentin/exp OR '1 (aminomethyl) cyclohexaneacetic acid' OR '2 [1 (aminomethyl) cyclohexyl] acetic acid' OR 'ci 945' OR 'ci945' OR 'dineurin' OR 'dm 1796' OR 'dm 5689' OR 'dm1796' OR 'dm5689' OR 'gabalept' OR 'gabaliquid geriasan' OR 'gabapen' OR 'gabapentin' OR 'gabatin' OR 'gantin' OR 'go 3450' OR 'go3450' OR 'goe 3450' OR 'goe3450' OR 'gralise' OR 'kaptin' OR 'keneil' OR 'neurontin' OR 'neurotonin' OR 'nupentin' OR 'sefelsa' OR 'serada') OR (perampanel/exp OR '2 (1, 6 dihydro 6 oxo 1 phenyl 3, 2` bipyridin 5 yl) benzonitrile' OR '2 (2 oxo 1 phenyl 5 pyridin 2 yl 1, 2 dihydro 3 pyridinyl) benzonitrile' OR '2 (2 oxo 1 phenyl 5 pyridin 2 yl 1, 2 dihydro 3 pyridyl) benzonitrile' OR '2 (2 oxo 1 phenyl 5 pyridin 2 yl 1, 2 dihydropyridin 3 yl) benzonitrile' OR '2 (2 oxo 1 phenyl 5 pyridin 2 ylpyridin 3 yl) benzonitrile' OR '2 [6` oxo 1` phenyl 1`, 6` dihydro (2, 3` bipyridin) 5` yl] benzonitrile' OR '3 (2 cyanophenyl) 5 (2 pyridinyl) 1 phenyl 1, 2 dihydropyridin 2 one' OR '5` (2 cyanophenyl) 1` phenyl 2, 3` bipyridinyl 6` (1`h) one' OR 'e 2007' OR 'e2007' OR 'er 155055 90' OR 'er 15505590' OR 'er15505590' OR 'fycompa' OR 'perampanel') OR (vigabatrin/exp OR '3 amino 5 carboxyhexene' OR '4 amino 4 ethenylbutyric acid' OR '4 amino 4 vinylbutanoic acid' OR '4 amino 4 vinylbutyric acid' OR '4 amino 5 hexenoic acid' OR '4 aminobutyric acid, 4 ethenyl' OR '4 aminohex 5 enoic acid' OR '4 vinyl 4 aminobutyric acid' OR '4 vinylaminobutyric acid' OR '4 vinylgaba' OR 'cpp 109' OR 'cpp109' OR 'gamma vinyl 4 aminobutyric acid' OR 'gamma vinyl gaba' OR 'gamma vinyl gamma aminobutyric acid' OR 'gamma vinylgaba' OR 'kigabeq' OR 'm 071754' OR 'm071754' OR 'mdl 71, 754' OR 'mdl 71754' OR 'n vinyl 4 aminobutyric acid' OR 'n vinyl gaba' OR 'n vinyl gamma aminobutyric acid' OR 'rmi 71754' OR 'rmi 71890' OR 'sabril' OR 'sabrilex' OR 'vigabatrin' OR 'vigadrone') OR (retigabine/exp OR '2 amino 1 ethoxycarbonylamino 4 (4 fluorobenzylamino) benzene' OR 'awd 21 360' OR 'awd 21-360' OR 'awd21 360' OR 'awd21-360' OR 'd 20443' OR 'd 23129' OR 'd20443' OR 'd23129' OR 'ethyl 2 amino 4 [ (para fluorobenzyl) amino] carbanilate' OR 'ethyl n [2 amino 4 [ (4 fluorophenyl) methylamino] phenyl] carbamate' OR 'ezogabine' OR 'gke 841' OR 'gke841' OR 'gw 582892x' OR 'gw582892x' OR 'n [2 amino 4 (4 fluorobenzylamino) phenyl] carbamic acid ethyl ester' OR 'n [2 amino 4 [ [(4 fluorophenyl) methyl] amino] phenyl] carbamic acid ethyl ester' OR 'potiga' OR 'potiva' OR 'retigabine' OR 'retigabine dihydrochloride' OR 'retigabine hydrochloride' OR 'trobalt' OR 'way 143841' OR 'way143841' OR 'xen 496' OR 'xen496') OR (carisbamate/exp OR '2 (2 chlorophenyl) 2 hydroxyethyl carbamate' OR '2 carbamoyloxy 1 (2 chlorophenyl) ethanol' OR '2 o carbamoyl 1 (2 chlorophenyl) 1, 2 ethanediol' OR 'carbamic acid 2 (2 chlorophenyl) 2 hydroxyethyl ester' OR 'carisbamate' OR 'rwj 333369' OR 'rwj333369' OR 'ykp 509' OR 'ykp509')) AND (('randomized controlled trial'/exp OR 'controlled trial, randomized' OR 'randomised controlled study' OR 'randomised controlled trial' OR 'randomized controlled study' OR 'randomized controlled trial' OR 'trial, randomized controlled') OR ('double blind procedure'/exp OR 'double blind clinical trial' OR 'double blind comparison' OR 'double blind design' OR 'double blind procedure' OR 'double blind studies' OR 'double blind study' OR 'double blind test' OR 'double blind trial' OR 'double masked clinical study' OR 'double masked clinical trial' OR 'double masked comparison' OR 'double masked design' OR 'double masked method' OR 'double masked procedure' OR 'double masked study' OR 'double masked test' OR 'double masked trial' OR 'double-blind clinical study' OR 'double-blind method'))

**PICO analysis in Cochrane database**

“focal epilepsy”, “partial epilepsy” AND “lacosamide”, “eslicarbazepine acetate”, “perampanel”, “brivaracetam”, “cenobamate”, “pregabalin”, “tiagabaine”, “vigabatrin”, “zonisamide”, “levetiracetam”, “oxcarbazepine”, “topiramate”, “lamotrigine”, “carbamazepine”, “valproate”, “rufinamide”, “gabapentin”, “retigabine”, “carisbamate”

**ClinicalTrials.gov**

“focal epilepsy”, “partial epilepsy” AND “lacosamide”, “eslicarbazepine acetate”, “perampanel”, “brivaracetam”, “cenobamate”, “pregabalin”, “tiagabaine”, “vigabatrin”, “zonisamide”, “levetiracetam”, “oxcarbazepine”, “topiramate”, “lamotrigine”, “carbamazepine”, “valproate”, “rufinamide”, “gabapentin”, “retigabine”, “carisbamate”

**Supplementary tables**

**Table S1: Baseline characteristics of studies included**

| Study. No | Author and year | Title | Trial arms | | | Age | | Time since diagnosis (years) | Seizure type, n (%) | Number of ASMs at baseline |
| --- | --- | --- | --- | --- | --- | --- | --- | --- | --- | --- |
|  |  |  | Intervention | Dose | Study population (n) |  |  |  |  |  |
|  |  |  |  |  |  |  |  |  |  |  |
|  |  |  |  |  |  |  |  |  |  |  |
| Perampanel | | | | | | | | | | |
| 1 | Nishida et al., 2017^1^ | Adjunctive perampanel in partial-onsetseizures: Asia-Pacific,randomized phase III study | Perampanel | 4 mg | 176 | ≥12 years | | 17.4 (11.1) | SPS, CPS, SGS | upto 4 |
|  |  |  |  | 8 mg | 175 |  |  | 16.9 (11.5) |  |  |
|  |  |  |  | 12 mg | 180 |  |  | 17.4 (11.2) |  |  |
|  |  |  | Placebo |  | 176 |  |  | 17.5 (10.9) |  |  |
| 2 | French JA et al., 2013^2^ | Evaluation of adjunctive perampanel in patients with refractory partial-onset seizures: Results of randomized global phase III study 305 | Perampanel | 8 mg/day | 129 | ≥12 years | | 270.3 (163.4) | SPS, CPS, SGS | up to 3 |
|  |  |  |  | 12 mg/day | 121 |  |  | 255.9 (158.6) |  |  |
|  |  |  | Placebo |  | 136 |  |  | 264.2 (155.3) |  |  |
| 3 | French JA et al., 2012^3^ | Adjunctive perampanel for refractory partial-onset seizures Randomized phase III study 304 | Perampanel | 8 mg/day | 133 | ≥12 years | | 282.8 (162.2) | SPS, CPS, SGS | up to 3 |
|  |  |  |  | 12 mg/day | 134 |  |  | 279.5 (172.4) |  |  |
|  |  |  | Placebo |  | 121 |  |  | 289.6 (154.4) |  |  |
| 4 | Krauss GL et al., 2012^4^ | Randomized phase III study 306 Adjunctive perampanel for refractory partial-onset seizures | Perampanel | 2 mg/day | 180 | ≥12 years | | 232.4 (145.2) | SPS, CPS, SGS | up to 3 |
|  |  |  |  | 4 mg/day | 172 |  |  | 236.9 (145.3) |  |  |
|  |  |  |  | 8 mg/day | 169 |  |  | 239.4 (142.9) |  |  |
|  |  |  | Placebo |  | 185 |  |  | 209.9 (128.1) |  |  |
| Rufinamide | | | | | | | | | | |
| 5 | Biton V et al., 2011^5^ | A randomized, double-blind, placebo-controlled, parallel-group study of rufinamide as adjunctive therapy for refractory partial-onset seizures | Rufinamide | 1,600 mg BID | 176 | 12– 80 years | | 10 | SPS, SGS | up to 3 |
|  |  |  | Placebo |  | 181 |  |  | 10 |  |  |
| 6 | Elger CE et al., 2010^6^ | A 24-week multicenter, randomized, double-blind, parallel-group, dose-ranging study of rufinamide in adults and adolescents with inadequately controlled partial seizures | Rufinamide | 400 mg/day | 125 | 15-65 years | | NA | SPS, CPS, SGS | up to 3 |
|  |  |  |  | 800 mg/day | 129 |  |  |  |  |  |
|  |  |  |  | 1600 mg/day | 133 |  |  |  |  |  |
|  |  |  | Placebo |  | 133 |  |  |  |  |  |
| 7 | Brodie M.J. et al., 2009^7^ | Rufinamide for the adjunctive treatment of partial seizures in adults and adolescents: A randomized placebo-controlled trial | Rufinamide | 800-3200 mg/day | 156 | ≥16 years | | NA | SGS | up to 2 |
|  |  |  | Placebo |  | 157 |  |  |  |  |  |
| Lamotrigine | | | | | | | | | | |
| 8 | Loiseau P et al., 1990^8^ | A randomised double-blind placebo-controlled crossover add-on trial of lamotrigine in patients with treatment-resistant partial seizures | Lamotrigine/placebo | 300 mg/day for patients on enzyme inducing drugs  150 mg/day for inhibited' patients (on valproate) | 10 | 16-65 years | | 17 | SPS, CPS | up to 2 |
|  |  |  | Placebo/lamotrigine |  | 13 |  |  | 17.8 |  |  |
| 9 | Naritoku DK et al., 2007^9^ | Lamotrigine extended-release as adjunctive therapy for partial seizures | Lamotrigine | based on adjunctive AED, the dose ranged from 200 to 500 mg/day | 118 | ≥12 years | | 21.8 (13.2) | SPS, CPS, SGS | NA |
|  |  |  | Placebo |  | 121 |  |  | 22.1 (16.1) |  |  |
| 10 | Smith.D et al.,1993^10^ | Outcomes of Add-on Treatment with Lamotrigine in Partial Epilepsy | Lamotrigine | 400 mg | 35 | 12-70 years | | 21 (range 4-45) | SPS, CPS, SGS | up to 2 |
|  |  |  | Placebo |  | 38 |  |  |  |  |  |
| 11 | Matsuo et al., 1993^11^ | Placebo-controlled study of the efficacy and safety of lamotrigine in patients with partial seizures. U.S. Lamotrigine Protocol 0.5 Clinical Trial Group | Lamotrigine | 300 mg/day | 71 | 18–65 years | | NA | SPS, SGS | up to 3 |
|  |  |  |  | 500 mg/day | 72 |  |  |  |  |  |
|  |  |  | Placebo |  | 73 |  |  |  |  |  |
| 12 | Messenheimer J et al., 1994^12^ | Lamotrigine therapy for partial seizures: a multicenter, placebo-controlled, double-blind, cross-over trial | Lamotrigine/placebo | <400 mg/day | 44 | 18–65 years | | 22.3 | CPS | up to 3 |
|  |  |  | Placebo/Lamotrigine |  | 44 |  |  | 24 |  |  |
| Pregabalin | | | | | | | | | | |
| 13 | French JA et al., 2003^13^ | Dose-response trial of pregabalin adjunctive therapy in patients with partial seizures | Pregabalin | 150 mg/day | 86 | 12-70 years | | NA | SGS | up to 3 |
|  |  |  |  | 300 mg/day | 90 |  |  |  |  |  |
|  |  |  | Placebo |  | 100 |  |  |  |  |  |
| 14 | French J et al., 2014^14^ | Adjunctive use of controlled-release pregabalin in adults with treatment-resistant partial seizures: A double-blind, randomized, placebo-controlled trial | Pregabalin | 165 mg/day | 113 | ≥18 years | | NA | SPS, CPS, SGS | up to 4 |
|  |  |  |  | 330 mg/day | 100 |  |  |  |  |  |
|  |  |  | Placebo |  | 110 |  |  |  |  |  |
| 15 | Lee BI et al., 2009^15^ | Pregabalin add-on therapy using a flexible, optimized dose schedule in refractory partial epilepsies: A double-blind, randomized, placebo-controlled, multicenter trial | Pregabalin | 150 mg/day to 600 mg/day | 119 | ≥18 years | | 16.5 | SPS, CPS, SGS | up to 4 |
|  |  |  | Placebo |  | 59 |  |  |  |  |  |
| 16 | Elger et al., 2005^16^ | Pregabalin Add-on Treatment in Patients with Partial Seizures: A Novel Evaluation of Flexible-dose and Fixed-dose Treatment in a Double-blind, Placebo-controlled Study | Pregabalin | Flexible Dose, 150-600 mg/day | 131 | ≥18 years | | 25.5 ±12.8 | SGS | up to 4 |
|  |  |  |  | Fixed Dose, 600 mg/day | 137 |  |  | 25.6 ± 13.3 |  |  |
|  |  |  | Placebo |  | 73 |  |  | 24.1 ±15.6 |  |  |
| 17 | Beydoun et al., 2005^17^ | Safety and efficacy of two pregabalin regimens for add-on treatment of partial epilepsy | Pregabalin | 300 mg BID | 103 | ≥18 years | | 25.9 ±12.5 | SPS, CPS, SGS | up to 4 |
|  |  |  |  | 200 mg TID | 111 |  |  | 27.7 ±13.4 |  |  |
|  |  |  | Placebo |  | 98 |  |  | 23.5 ± 11.9 |  |  |
| 18 | French et al., 2016^18^ | Adjunctive pregabalin vs gabapentin for focal seizures Interpretation of comparative outcomes | Pregabalin | 300-600 mg/day | 241 | 18–80 years | | 15.6 (0.7–49.2) | SPS, CPS, SGS | up to 4 |
|  |  |  | Gabapentin | 1200-1800 mg/day | 241 |  |  | 15.8 (1.8–52.9) |  |  |
| 19 | Zaccara et al., 2014^19^ | Efficacy and safety of pregabalin versus levetiracetam as adjunctive therapy in patients with partial seizures: A randomized, double-blind, noninferiority trial | Pregabalin | 300 mg BID | 254 | ≥18 years | | 15.5 (2.0–52.8) | SPS, CPS, SGS | up to 2 |
|  |  |  | levetiracetam | 1500 mg BID | 255 |  |  | 17.3 (1.9–59.6) |  |  |
| 20 | Arroyo et al., 2004^20^ | Pregabalin Add-on Treatment: A Randomized, Double-blind, Placebo-controlled, Dose-Response Study in Adults with Partial Seizures | Pregabalin | 150 mg/day | 99 | ≥18 years | | 24.8 (12.65) | SPS, CPS, SGS | up to 3 |
|  |  |  | Placebo |  | 96 |  |  | 22.78 (13.58) |  |  |
| Tiagabine | | | | | | | | | | |
| 21 | Uthman et al., 1998^21^ | Tiagabine for complex partial seizures. A Randomized, Add-on, Dose-Response Trial | Tiagabine | 32 mg/day | 86 | 12-77 years | | NA | CPS | up to 3 |
|  |  |  |  | 56 mg/day | 55 |  |  |  |  |  |
|  |  |  | Placebo |  | 90 |  |  |  |  |  |
| 22 | Kälviäinen R et al., 1998^22^ | A double-blind, placebo-controlled trial of tiagabine given three-times daily as add-on therapy for refractory partial seizures | Tiagabine | 12- 30 mg/day  (10 mg TID) | 77 | 16-75 years | | 24.9 (2–52) | SPS, CPS, SGS | 1 |
|  |  |  | Placebo |  | 77 |  |  | 23 (1–49) |  |  |
| 23 | Sachdeo RC et al.,1997^23^ | Tiagabine Therapy for Complex Partial Seizures, A Dose-Frequency Study | Tiagabine | 16 mg BID | 106 | 12-75 years | | 18 | SPS, CPS | NA |
|  |  |  |  | 8 mg, FID | 105 |  |  | 22 |  |  |
|  |  |  | Placebo |  | 107 |  |  | 24 |  |  |
| 24 | Richens et al., 1995^24^ | Adjunctive treatment of partial seizures with tiagabine: A placebo-controlled trial | Tiagabine | 52 mg/day | 94 | 18–65 years | | 23 | SPS, CPS, SGS | NA |
|  |  |  | Placebo |  |  |  |  |  |  |  |
| Vigabatrin | | | | | | | | | | |
| 25 | Griinewald RA et al., 1994^25^ | Effects of vigabatrin on partial seizures and cognitive function | Vigabatrin | 1.5 g BID | 22 | 15-61 years | | NA | SPS, CPS, SGS | NA |
|  |  |  | Placebo |  | 23 |  |  |  |  |  |
| 26 | Brodie MJ et al., 1999^26^ | Double-blind substitution of vigabatrin and valproate in carbamazepine-resistant partial epilepsy | Vigabatrin | 3 g/day | 108 | 12-75 years | | NA | SPS, CPS, SGS | NA |
|  |  |  | Valproate | 1.5 g/day | 107 |  |  |  |  |  |
| 27 | Beran RG et al., 1996^27^ | A double-blind, placebo-controlled crossover study of vigabatrin 2 g/day and 3 g/day in uncontrolled partial seizures | Vigabatrin 2 g | 2 g/day | 20 | 16-65 years | | NA | CPS | up to 3 |
|  |  |  | Vigabatrin 3 g | 3 g/day | 20 |  |  |  |  |  |
|  |  |  | Placebo 2 g |  | 22 |  |  |  |  |  |
|  |  |  | Placebo 3 g |  | 18 |  |  |  |  |  |
| 28 | Dean et al., 1998^28^ | Dose-Response Study of Vigabatrin as Add-on Therapy in Patients with Uncontrolled Complex Partial Seizures | Vigabatrin | 3 g/day | 43 | 18–60 years | | 20 ± 9 | SPS, CPS, SGS | up to 3 |
|  |  |  | Placebo |  | 45 |  |  | 22 ± 11 |  |  |
| 29 | French et al., 1996^29^ | A double-blind, placebo-controlled study of Vigabatrin three g/day in patients with uncontrolled complex partial seizures | Vigabatrin | 3 g/day | 92 | 18–60 years | | NA | CPS,  SGS | up to 2 |
|  |  |  | Placebo |  | 90 |  |  |  |  |  |
| Gabapentin | | | | | | | | | | |
| 30 | H. Anhut et al.,1994^30^ | Gabapentin (Neurontin) as Add-on Therapy in Patients Placebo-Controlled Study | Gabapentin | 900 mg/day | 111 | ≥12 years | | NA | SPS, CPS, SGS | up to 2 |
|  |  |  |  | 1200 mg/day | 52 |  |  |  |  |  |
|  |  |  | Placebo |  | 109 |  |  |  |  |  |
| 31 | Leach JP et al., 1997^31^ | Gabapentin and cognition: A double blind, dose ranging, placebo-controlled study in refractory epilepsy | Gabapentin | 400 mg, 600 mg, and 800 mg TID | 14 | 16-67 years | | NA | SPS, CPS, SGS |  |
|  |  |  | Placebo |  | 13 |  |  |  |  |  |
| 32 | The US Gabapentin Study Group No. 5, 1993^32^ | Gabapentin as add-on therapy in refractory partial epilepsy: a double-blind, placebo-controlled, parallel-group study. The US Gabapentin Study Group No. 5 | Gabapentin | 1200 mg/day | 101 | ≥16 years | | NA | SPS, CPS, SGS | up to 3 |
|  |  |  |  | 1800 mg/day | 54 |  |  |  |  |  |
|  |  |  | Placebo |  | 98 |  |  |  |  |  |
| 33 | Yamauchi T et al., 2006^33^ | Treatment of partial seizures with gabapentin: Double-blind, placebo-controlled, parallel-group study | Gabapentin | 1200 mg/day | 86 | ≥16 years | | NA | SPS, CPS, SGS | up to 2 |
|  |  |  |  | 1800 mg/day | 41 |  |  |  |  |  |
|  |  |  | Placebo |  | 82 |  |  |  |  |  |
| 34 | UK Gabapentin Study Group, 1990^34^ | Gabapentin in partial epilepsy | Gabapentin | 1200 mg/day | 61 | 14-73 years | | 17 (2-47) | SPS, CPS, SGS | up to 3 |
|  |  |  | Placebo |  | 66 |  |  |  |  |  |
| Zonisamide | | | | | | | | | | |
| 35 | Sackellares JC et al., 2004^35^无responder rate | Randomized Controlled clinical trial of Zonisamide as adjunctive treatment for refractory partial seizures | Zonisamide | <400 mg/day or ≥400 mg/day | 78 | 17 - 65 years | | NA | SPS, CPS, SGS | up to 2 |
|  |  |  | Placebo |  | 74 |  |  |  |  |  |
| 36 | E. Faught et al., 2001^36^ | Randomized controlled trial of zonisamide for the treatment of refractory partial-onset seizures | Zonisamide-B1 | 400 mg/day  100 mg/d of zonisamide for weeks 1 through 5, 200 mg during week 6, 300 mg during week 7, and 400 mg for the final 13 weeks (weeks 8 through 20). | 60 | ≥12 years | | NA | SPS, CPS, SGS | NA |
|  |  |  | Zonisamide-B2 | 100 mg/d of zonisamide for the first week, 200 mg/d over weeks 2 through 6, 300 mg/d for week 7, and 400 mg/d for weeks 8 through 20. | 58 |  |  |  |  |  |
|  |  |  | Placebo | Placebo for 12 weeks. At week 13, crossed over to zonisamide 400 mg/day | 85 |  |  |  |  |  |
| 37 | Brodie MJ et al., 2005^37^ | Dose-dependent Safety and Efficacy of Zonisamide: A Randomized, Double-blind, Placebo-controlled Study in Patients with Refractory Partial Seizures | Zonisamide | 100 mg/day | 56 | ≥12 years | | 279.5 | SPS, CPS, SGS | up to 4 |
|  |  |  |  | 300 mg/day | 55 |  |  | 188 |  |  |
|  |  |  |  | 500 mg/day | 118 |  |  | 227 |  |  |
|  |  |  | Placebo |  | 120 |  |  | 254 |  |  |
| 38 | Lu et al., 2011^38^ | Efficacy and safety of adjunctive zonisamide in adult patients with refractory partial-onset epilepsy: A randomized, double-blind, placebo-controlled trial | Zonisamide | 300 or 400 mg/day | 52 | 18-70 years | |  | SPS, CPS, SGS | up to 3 |
|  |  |  | Placebo |  | 50 |  |  |  |  |  |
| 39 | Schmidt et al., 1993^39^ | Zonisamide for add-on treatment of refractory partial epilepsy: a European double-blind trial | Zonisamide | 1.5-6 mg/kg/day | 71 | 18–59 years | | 23.5 | CPS | - |
|  |  |  | Placebo |  | 68 |  |  | 20.9 |  |  |
| 40 | Brodie M.J, 2004^40^ | Zonisamide clinical trials: European experience | Zonisamide | 400 mg/day | 73 | 18–59 years | | NA | SPS, CPS, SGS | up to 3 |
|  |  |  | Placebo |  | 71 |  |  |  |  |  |
| Brivaracetam | | | | | | | | | | |
| 41 | Ryvlin P et al., 2014^41^ | Adjunctive brivaracetam in adults with uncontrolled focal epilepsy: Results from a double-blind, randomized, placebo-controlled trial | Brivaracetam | 50 mg/day | 99 | 16–70 years | | NA | SPS, CPS, SGS | up to 3 |
|  |  |  |  | 100 mg/day | 100 |  |  |  |  |  |
|  |  |  | Placebo |  | 100 |  |  |  |  |  |
| 42 | Klein P et al., 2015^42^ | A randomized, double-blind, placebo-controlled, multicenter, parallel-group study to evaluate the efficacy and safety of adjunctive brivaracetam in adult patients with uncontrolled partial-onset seizures | Brivaracetam | 100 mg/day | 253 | ≥16–80 years | | NA | SPS, CPS, SGS | up to 3 |
|  |  |  |  | 200 mg/day | 250 |  |  |  |  |  |
|  |  |  | Placebo |  | 261 |  |  |  |  |  |
| 43 | Biton V et al., 2014^43^ | Brivaracetam as adjunctive treatment for uncontrolled partial epilepsy in adults: A phase III randomized, double-blind, placebo-controlled trial | Brivaracetam | 50 mg/day | 101 | 16–70 years | | NA | SPS, CPS, SGS | up to 3 |
|  |  |  | Placebo |  | 98 |  |  |  |  |  |
| Lacosamide | | | | | | | | | | |
| 44 | Chung S et al., 2010^44^ | Lacosamide as adjunctive therapy for partial-onset seizures: A randomized controlled trial | Lacosamide | 400 mg/day | 204 | 16–70 years | | 24.5 (13.16) | CPS, SGS | up to 3 |
|  |  |  | Placebo |  | 104 |  |  | 25.4 (13.34) |  |  |
| 45 | Hong Z et al., 2016^45^ | Efficacy and safety of adjunctive lacosamide for the treatment of partial-onset seizures in Chinese and Japanese adults: A randomized, double-blind, placebo-controlled study | Lacosamide | 200 mg/day  (100 mg BID) | 183 | 16-70 years | | 18.3 (10.9) | SPS, CPS, SGS | up to 3 |
|  |  |  |  | 400 mg/day  (200 mg BID) | 180 |  |  | 17.9 (11.7) |  |  |
|  |  |  | Placebo |  | 184 |  |  | 16.8 (11.5) |  |  |
| 46 | Halász P et al., 2009^46^ | Adjunctive lacosamide for partial-onset seizures: Efficacy and safety results from a randomized controlled trial | Lacosamide | 200 mg/day | 163 | 16–70 years | | 22.9 ± 12.30 | SPS, CPS, SGS | up to 3 |
|  |  |  |  | 400 mg/day | 159 |  |  | 22.8 ± 13.15 |  |  |
|  |  |  | Placebo |  | 163 |  |  | 21.1 ± 12.23 |  |  |
| 47 | Menachem et al., 2007^47^ | Efficacy and Safety of Oral Lacosamide as Adjunctive Therapyin Adults with Partial-Onset Seizures | Lacosamide | 200 mg/day | 107 | 18–65 years | | 25.1 ± 12.89 | SPS, CPS, SGS | - |
|  |  |  |  | 400 mg/day | 108 |  |  | 24.7 ± 13.08 |  |  |
|  |  |  | Placebo |  | 97 |  |  | 24.6 ± 11.77 |  |  |
| Levetiracetam | | | | | | | | | | |
| 48 | Peltola J et al., 2009^48^ | Once-daily extended-release levetiracetam as adjunctive treatment of partial-onset seizures in patients with epilepsy: A double-blind, randomized, placebo-controlled trial | Levetiracetam | 1,000 mg/day | 79 | 12– 70 years | | 20.86 ± 15.18 | SPS, CPS | up to 4 |
|  |  |  | Placebo |  | 79 |  |  | 15.95 ± 11.51 |  |  |
| 49 | Inoue and et al., 2015^49^ | Efficacy and tolerability of levetiracetam as adjunctive therapy in Japanese patients with uncontrolled partial-onset seizures | Levetiracetam | 1000 mg/day | 70 | 16-65 years | | NA | SGS | up to 3 |
|  |  |  |  | 2000 mg/day | 70 |  |  |  |  |  |
|  |  |  |  | 3000 mg/day | 70 |  |  |  |  |  |
|  |  |  | Placebo |  | 70 |  |  |  |  |  |
| 50 | Wu X.-Y et al., 2009^50^ | Multicenter double-blind, randomized, placebo-controlled trial of levetiracetam as add-on therapy in Chinese patients with refractory partial-onset seizures | Levetiracetam | 3000 mg/day | 102 | 16–70 years | | NA | SPS, CPS, SGS | NA |
|  |  |  | Placebo |  | 100 |  |  |  |  |  |
| 51 | Xiao Z et al., 2009^51^ | Efficacy and safety of levetiracetam (3,000 mg/day) as an adjunctive therapy in Chinese patients with refractory partial seizures | Levetiracetam | 3000 mg/day | 28 | 16–70 years | NA | | SPS, CPS, SGS | NA |
|  |  |  | Placebo |  | 28 | 16–70 years | | NA |  |  |
| 52 | Zhou B et al., 2008^52^ | Effects of levetiracetam as an add-on therapy on cognitive function and quality of life in patients with refractory partial seizures | Levetiracetam | 1500 mg BID | 13 | 16–70 years | | NA | SPS, CPS, SGS | up to 2 |
|  |  |  | Placebo |  | 11 |  |  |  |  |  |
| 53 | Tsai JJ et al., 2006^53^ | Efficacy and safety of levetiracetam (up to 2000 mg/day) in Taiwanese patients with refractory partial seizures: A multicenter, randomized, double-blind, placebo-controlled study | Levetiracetam | 1000 mg/day | 47 | 16–70 years | | NA | Partial, primary Seizure, SGS | up to 4 |
|  |  |  | Placebo |  | 47 |  |  |  |  |  |
| 54 | Boon P et al., 2002^54^ | Dose-response effect of levetiracetam 1000 and 2000 mg/day in partial epilepsy | Levetiracetam-placebo sequence | 1000 mg/day | 53 | 16-65 years | | NA | SPS, CPS, SGS | up to 3 |
|  |  |  | Levetiracetam 1000 mg/day-Levetiracetam 2000 mg/day sequence | 1000 mg/day-levetiracetam 2000 mg/day sequence | 53 |  |  |  |  |  |
|  |  |  | Levetiracetam 2000 mg/day-placebo sequence | 2000 mg/day | 54 |  |  |  |  |  |
|  |  |  | Levetiracetam 2000 mg/day-Levetiracetam 1000 mg/day sequence | 2000 mg/day-levetiracetam 1000 mg/day sequence | 52 |  |  |  |  |  |
|  |  |  | Placebo-levetiracetam 1000 mg/day sequence | | 58 |  |  |  |  |  |
|  |  |  | Placebo-levetiracetam 2000 mg/day sequence | | 54 |  |  |  |  |  |
| 55 | Shorvon S.D et al., 2000^55^ | Multicenter double-blind, randomized, placebo-controlled trial of levetiracetam as add-on therapy in patients with refractory partial seizures | Levetiracetam | 1000 mg/d | 106 | 16-65 years | | NA | SPS, CPS, SGS | up to 3 |
|  |  |  |  | 2000 mg/d | 106 |  |  |  |  |  |
|  |  |  | Placebo |  | 112 |  |  |  |  |  |
| 56 | Cereghino J.J. et.al, 2000^56^ | Levetiracetam for partial seizures: Results of a double-blind, randomized clinical trial | Levetiracetam | 1000 mg/d | 98 | 16–70 years | | NA | US | up to 3 |
|  |  |  |  | 3000 mg/d | 101 |  |  |  |  |  |
|  |  |  | Placebo |  | 95 |  |  |  |  |  |
| 57 | Betts T et al., 2000^57^ | A multicentre, double-blind, randomized, parallel group study to evaluate the tolerability and efficacy of two oral doses of levetiracetam, 2000 mg daily and 4000 mg daily, without titration in patients with refractory epilepsy | Levetiracetam | 2000 mg/d | 42 | 16–70 years | | 21.1 ± 14.4 | GS | NA |
|  |  |  | Placebo |  | 39 |  |  | 26 ± 13.2 |  |  |
| 58 | Ben-Menachem et al., 2000^58^ | Efficacy and tolerability of levetiracetam 3000 mg/d in patients with refractory partial seizures: A multicenter, double-blind, responder-selected study evaluating monotherapy | Levetiracetam | 1500 mg BID | 181 | 16-70 years | | 19 (11) | SPS, CPS, SGS | 1 |
|  |  |  | Placebo |  | 105 |  |  | 19 (12) |  |  |
| Retigabine | | | | | | | | | | |
| 59 | Lim KS et al., 2016^59^ | Efficacy and safety of retigabine/ezogabine as adjunctive therapy in adult Asian patients with drug-resistant partial-onset seizures: A randomized, placebo-controlled Phase III study | Retigabine | 600 mg/day (200 mg TID) | 26 | ≥18 years | | NA | SGS | up to 3 |
|  |  |  | Placebo |  | 25 |  |  |  |  |  |
| 60 | Brodie MJ et al., 2010^60^ | Efficacy and safety of adjunctive ezogabine (retigabine) in refractory partial epilepsy | Retigabine | 600 mg/d [200 mg TID] | 181 | 18-75 years | | 22.5 (13.0) | PS | - |
|  |  |  | Placebo | | 179 |  |  | 22.8 (11.8) |  |  |
| 61 | Porter RJ et al., 2007^61^ | Randomized, multicenter, dose-ranging trial of retigabine for partial-onset seizures | Retigabine | 600 mg/day | 99 | 16-70 years | | 21.2 (12.0) | SPS, CPS, SGS | up to 3 |
|  |  |  | Placebo |  | 96 |  |  | 20.8 (11.2) |  |  |
| 62 | French JA et al., 2011^62^ | Randomized, double-blind, placebo-controlled trial of ezogabine (retigabine) in partial epilepsy | Retigabine | 1,200 mg/day | 154 | 18-75 years | | 23.7 (13.0) | SPS,  CPS | up to 3 |
|  |  |  | Placebo |  | 152 |  |  | 23.1 (12.8) |  |  |
| Cenobamate | | | | | | | | | | |
| 63 | Krauss GL et al., 2020^63^ | Safety and efficacy of adjunctive cenobamate (YKP3089) in patients with uncontrolled focal seizures: a multicentre, double-blind, randomised, placebo-controlled, dose-response trial | Cenobamate | 200 mg | 110 | 18-70 years | | 22.8 (13.2) | SPS, CPS, SGS | up to 4 |
|  |  |  |  | 400 mg | 111 |  |  | 24.4 (14.2) |  |  |
|  |  |  | Placebo |  | 108 |  |  | 23.0 (14.2) |  |  |
| Oxcarbazepine | | | | | | | | | | |
| 64 | French JA et al., 2014^64^ | Efficacy and safety of extended-release oxcarbazepine (Oxtellar XR™) as adjunctive therapy in patients with refractory partial-onset seizures: A randomized controlled trial | Oxcarbazepine | 1200 mg | 122 | 18–65 years | | 21.3 (14.5) | SPS | up to 3 |
|  |  |  | Placebo |  | 121 |  | | 21.2 (13.9) |  |  |
| 65 | Barcs G et al., 2000^65^ | Oxcarbazepine Placebo-Controlled, Dose-Ranging Trial in Refractory Partial Epilepsy | Oxcarbazepine | 600 mg/day | 169 168 | 15-65 years | |  | SGS | up to 4 |
|  |  |  |  | 1200 mg/day | 178 177 |  |  |  |  |  |
|  |  |  | Placebo |  | 173 |  |  |  |  |  |
| Eslicarbazepine | | | | | | | | | | |
| 66 | Ben-Menachem et al., 2010^66^ | Eslicarbazepine acetate as adjunctive therapy in adult patients with partial epilepsy | Eslicarbazepine | 800 mg/day | 101 | ≥18 years | | 22.4 (11.63) | SPS, CPS, SGS | up to 4 |
|  |  |  |  | 1200 mg/day | 98 |  |  | 23.0 (12.90) |  |  |
|  |  |  | Placebo |  | 100 |  |  | 25.4 (13.06) |  |  |
| 67 | Gil-Nagel A et.al, 2009^67^ | Efficacy and safety of 800 and 1200 mg eslicarbazepine acetate as adjunctive treatment in adults with refractory partial-onset seizures | Eslicarbazepine | 800 mg/day | 85 | ≥18 years | | 22.5 (11.78) | SPS, CPS, SGS | up to 4 |
|  |  |  |  | 1200 mg/day | 80 |  |  | 23.0 (13.01) |  |  |
|  |  |  | Placebo |  | 87 |  |  | 23.8 (13.03) |  |  |
| 68 | Elger C et al., 2009^68^ | Efficacy and safety of eslicarbazepine acetate as adjunctive treatment in adults with refractory partial-onset seizures: A randomized, double-blind, placebo-controlled, parallel-group phase III study | Eslicarbazepine | 800 mg/day | 98 | ≥18 years | | 23.1 (13.50) | SPS, CPS, SGS | up to 3 |
|  |  |  |  | 1200 mg/day | 102 |  |  | 20.4 (11.85) |  |  |
|  |  |  | Placebo |  | 102 |  |  | 19.4 (12.57) |  |  |
| 69 | Sperling MR et al., 2015^69^ | Eslicarbazepine acetate as adjunctive therapy in patients with uncontrolled partial-onset seizures: Results of a phase III, double-blind, randomized, placebo-controlled trial | Eslicarbazepine | 800 mg/day | 216 | ≥16 years | | - | SPS,  CPS | up to 3 |
|  |  |  |  | 1200 mg/day | 210 |  |  |  |  |  |
|  |  |  | Placebo |  | 224 |  |  |  |  |  |
| 70 | Elger et al et al., 2007^70^ | Eslicarbazepine acetate: A double-blind, add-on, placebo-controlled exploratory trial in adult patients with partial-onset seizures | Eslicarbazepine | 1200 mg/day | 50 | 18–65 years | | 16.7 (11.7) | SPS, CPS, SGS | up to 2 |
|  |  |  |  | 600 mg BID | 46 |  |  | 19.5 (12.6), |  |  |
|  |  |  | Placebo |  | 47 |  |  | 20.0 (13.6) |  |  |
| Topiramate | | | | | | | | | | |
| 71 | Guberman et al., 2002^71^ | Low-dose topiramate in adults with treatment-resistant partial-onset seizures | Topiramate | 200 mg/day | 85 | 18–65 years | | 19 | SPS, CPS, SGS | up to 2 |
|  |  |  |  | 400 mg/day | 86 |  |  |  |  |  |
|  |  |  | Placebo |  | 92 |  |  | 18 |  |  |
| 72 | Chung et al., 2014^72^ | Once-daily USL255 as adjunctive treatment of partial-onset seizures: Randomized phase III study | Topiramate | 200 mg/day | 124 | 18-75 years | | 20.9 (13.7) | SPS, CPS, SGS | up to 3 |
|  |  |  | Placebo |  | 125 |  |  | 20 (13.1) |  |  |
| 73 | Yen et al., 2000^73^ | A Double-Blind, Placebo-Controlled Study of Topiramate in Adult Patients with Refractory Partial Epilepsy | Topiramate | 300 mg/day | 23 | 18–65 years | | 14.9 ± 10.9 | SPS, CPS | up to 4 |
|  |  |  | Placebo |  | 23 |  |  | 18.9 ± 11.1 |  |  |
| 74 | Sharief et al., 1996^74^ | Double-blind, placebo-controlled study of topiramate in patients with refractory partial epilepsy | Topiramate | 400 mg/day | 23 | 18–65 years | | NA | SPS, CPS, SGS | NA |
|  |  |  | Placebo |  | 24 |  |  |  |  |  |
| 75 | Faught et.al., 1996^75^ | Topiramate placebo-controlled dose ranging trial in refractory partial epilepsy using ZOO-, 400-, and 600 mg daily dosages | Topiramate | 200 mg/day | 45 | 18–65 years | | NA | SPS, CPS, SGS | up to 2 |
|  |  |  |  | 400 mg/day | 45 |  |  |  |  |  |
|  |  |  | Placebo |  | 45 |  |  |  |  |  |

BID, twice-daily; CPS, complex partial seizure; FID, four times daily; PS, Partial seizure; PPS, Partial primary seizure; SGS, secondary generalized seizures; SPS, simple partial seizure; TID, thrice-daily; US, Uncontrolled seizure, NA, not available

| Brivaracetam |  |  |  |  |  |  |  |  |  |  |  |  |  |  |  |  |  |
| --- | --- | --- | --- | --- | --- | --- | --- | --- | --- | --- | --- | --- | --- | --- | --- | --- | --- |
| 0.6223 (0.4155, 0.9179) | Cenobamate |  |  |  |  |  |  |  |  |  |  |  |  |  |  |  |  |
| 0.9554 (0.7228, 1.255) | 1.531 (1.032, 2.321) | Eslicarbazepine_  acetate |  |  |  |  |  |  |  |  |  |  |  |  |  |  |  |
| 0.6358 (0.4612, 0.872) | 1.017 (0.6724, 1.557) | 0.6678 (0.4904, 0.9095) | Gabapentin |  |  |  |  |  |  |  |  |  |  |  |  |  |  |
| 0.9652 (0.7306, 1.283) | 1.557 (1.05, 2.327) | 1.016 (0.7791, 1.314) | 1.527 (1.106, 2.068) | Lacosamide |  |  |  |  |  |  |  |  |  |  |  |  |  |
| 0.8204 (0.5491, 1.176) | 1.322 (0.8456, 2.09) | 0.863 (0.5891, 1.203) | 1.293 (0.8771, 1.9) | 0.854 (0.5866, 1.198) | Lamotrigine |  |  |  |  |  |  |  |  |  |  |  |  |
| 0.8671 (0.6717, 1.131) | 1.39 (0.9569, 2.05) | 0.9089 (0.7147, 1.163) | 1.362 (1.027, 1.85) | 0.8955 (0.7019, 1.148) | 1.056 (0.7604, 1.51) | Levetiracetam |  |  |  |  |  |  |  |  |  |  |  |
| 0.9411 (0.6634, 1.315) | 1.513 (0.9788, 2.36) | 0.985 (0.7039, 1.366) | 1.486 (1.044, 2.096) | 0.9747 (0.7079, 1.326) | 1.153 (0.7654, 1.72) | 1.085 (0.7913, 1.474) | Oxcarbazepine |  |  |  |  |  |  |  |  |  |  |
| 1.019 (0.7772, 1.345) | 1.632 (1.11, 2.5) | 1.066 (0.8326, 1.391) | 1.597 (1.179, 2.183) | 1.053 (0.8207, 1.353) | 1.238 (0.878, 1.796) | 1.177 (0.9294, 1.502) | 1.08 (0.7776, 1.491) | Perampanel |  |  |  |  |  |  |  |  |  |
| 1.78 (1.465, 2.209) | 2.861 (2.043, 4.131) | 1.864 (1.551, 2.272) | 2.794 (2.211, 3.586) | 1.842 (1.542, 2.211) | 2.165 (1.639, 3.035) | 2.061 (1.757, 2.407) | 1.893 (1.469, 2.484) | 1.749 (1.471, 2.102) | Placebo |  |  |  |  |  |  |  |  |
| 0.6302 (0.4539, 0.8739) | 1.01 (0.6723, 1.565) | 0.6594 (0.4872, 0.8861) | 0.9923 (0.8501, 1.147) | 0.6474 (0.4775, 0.8838) | 0.7689 (0.5206, 1.151) | 0.7295 (0.5366, 0.9646) | 0.6675 (0.4708, 0.9621) | 0.6181 (0.4542, 0.8443) | 0.3544 (0.274, 0.4437) | Pregabalin |  |  |  |  |  |  |  |
| 0.9409 (0.6491, 1.378) | 1.516 (0.9446, 2.522) | 0.9885 (0.6845, 1.417) | 1.474 (0.996, 2.224) | 0.978 (0.6655, 1.421) | 1.149 (0.7288, 1.788) | 1.085 (0.7578, 1.573) | 0.9983 (0.6614, 1.557) | 0.9267 (0.6399, 1.315) | 0.5293 (0.3805, 0.7322) | 1.489 (1.008, 2.242) | Retigabine_Ezogabine |  |  |  |  |  |  |
| 0.9808 (0.6775, 1.449) | 1.576 (1.011, 2.509) | 1.029 (0.7239, 1.485) | 1.538 (1.038, 2.263) | 1.014 (0.721, 1.471) | 1.197 (0.7988, 1.831) | 1.126 (0.8154, 1.586) | 1.035 (0.7093, 1.549) | 0.9668 (0.6909, 1.372) | 0.5488 (0.4101, 0.735) | 1.554 (1.058, 2.306) | 1.046 (0.6705, 1.602) | Rufinamide |  |  |  |  |  |
| 0.5338 (0.3037, 0.8971) | 0.8566 (0.4522, 1.538) | 0.5616 (0.3122, 0.937) | 0.8338 (0.4723, 1.443) | 0.553 (0.3144, 0.9184) | 0.649 (0.3415, 1.153) | 0.6146 (0.3437, 1.02) | 0.5639 (0.3152, 0.9647) | 0.5219 (0.2926, 0.8632) | 0.2997 (0.1745, 0.4828) | 0.8387 (0.4642, 1.48) | 0.5628 (0.2969, 1.012) | 0.5394 (0.3022, 0.9512) | Tiagabaine |  |  |  |  |
| 0.9209 (0.6684, 1.261) | 1.488 (0.9846, 2.276) | 0.9665 (0.701, 1.306) | 1.44 (1.025, 2.037) | 0.954 (0.6904, 1.286) | 1.12 (0.7777, 1.674) | 1.062 (0.7895, 1.418) | 0.9761 (0.6733, 1.402) | 0.9018 (0.6544, 1.221) | 0.5167 (0.4041, 0.6526) | 1.452 (1.048, 2.058) | 0.9761 (0.6546, 1.45) | 0.9377 (0.636, 1.348) | 1.74 (0.9924, 3.06) | Topiramate |  |  |  |
| 0.6026 (0.3856, 0.9441) | 0.9714 (0.5642, 1.619) | 0.629 (0.4017, 0.9689) | 0.9498 (0.6444, 1.367) | 0.6221 (0.4057, 0.9639) | 0.7295 (0.4461, 1.21) | 0.6895 (0.4515, 1.073) | 0.6372 (0.4031, 1.017) | 0.5951 (0.3738, 0.8993) | 0.337 (0.2254, 0.5001) | 0.9546 (0.6413, 1.403) | 0.6377 (0.3785, 1.062) | 0.6136 (0.3774, 0.9931) | 1.132 (0.6106, 2.166) | 0.6556 (0.4149, 1.024) | Valproate |  |  |
| 0.5901 (0.4055, 0.8426) | 0.9511 (0.5968, 1.49) | 0.6165 (0.4374, 0.8788) | 0.9304 (0.71, 1.219) | 0.6084 (0.4346, 0.8638) | 0.7165 (0.4727, 1.117) | 0.678 (0.4833, 0.9421) | 0.6256 (0.4207, 0.9125) | 0.5807 (0.4025, 0.8224) | 0.3296 (0.2428, 0.4381) | 0.9344 (0.6927, 1.253) | 0.6241 (0.401, 0.9708) | 0.5998 (0.3899, 0.9118) | 1.106 (0.6067, 2.029) | 0.6393 (0.4343, 0.923) | 0.9759 (0.7576, 1.264) | Vigabatrin |  |
| 0.9029 (0.6637, 1.24) | 1.458 (0.9734, 2.219) | 0.9508 (0.6999, 1.276) | 1.419 (1.015, 1.994) | 0.9373 (0.6883, 1.247) | 1.106 (0.7618, 1.626) | 1.044 (0.7855, 1.373) | 0.9639 (0.6664, 1.341) | 0.8908 (0.6618, 1.169) | 0.5092 (0.4013, 0.6288) | 1.435 (1.022, 2.02) | 0.9582 (0.6548, 1.413) | 0.9254 (0.6391, 1.339) | 1.702 (0.9977, 3.04) | 0.9824 (0.6977, 1.38) | 1.516 (0.9439, 2.371) | 1.537 (1.075, 2.254) | Zonisamide |

**Table S2.** Pairwise comparison of treatments associated with ≥50% responder rate

Comparisons should be read from left to right. The efficacy estimate is located at the intersection of the column-defining treatment and the row-defining treatment. An RR above 1 favours the column-defining treatment. To obtain RRs for comparisons in the opposing direction, reciprocals should be taken.

| Cenobamate |  |  |  |  |  |  |  |  |  |  |  |
| --- | --- | --- | --- | --- | --- | --- | --- | --- | --- | --- | --- |
| 1.117 (0.3046, 3.569) | Eslicarbazepine_acetate |  |  |  |  |  |  |  |  |  |  |
| 1.815 (0.04749, 20.47) | 1.643 (0.03802, 19.06) | Gabapentin |  |  |  |  |  |  |  |  |  |
| 1.834 (0.9398, 3.922) | 1.602 (0.6231, 6.09) | 0.9978 (0.1031, 38.95) | Lacosamide |  |  |  |  |  |  |  |  |
| 1.166 (0.5514, 2.754) | 1.047 (0.3799, 4.075) | 0.6485 (0.06735, 25.8) | 0.64 (0.3372, 1.197) | Levetiracetam |  |  |  |  |  |  |  |
| 1.318 (0.602, 3.049) | 1.186 (0.4099, 4.29) | 0.7187 (0.06993, 29.38) | 0.729 (0.3951, 1.323) | 1.118 (0.5522, 2.273) | Perampanel |  |  |  |  |  |  |
| 4.842 (2.791, 9.678) | 4.348 (1.803, 15.23) | 2.631 (0.2732, 114.1) | 2.659 (1.902, 3.899) | 4.156 (2.577, 7.058) | 3.684 (2.338, 6.213) | Placebo |  |  |  |  |  |
| 1.853 (0.0474, 20.46) | 1.685 (0.03686, 19.71) | 1.015 (0.7817, 1.309) | 0.9897 (0.02531, 9.636) | 1.561 (0.0393, 15.57) | 1.396 (0.03128, 14.65) | 0.3789 (0.008738, 3.678) | Pregabalin |  |  |  |  |
| 1.876 (0.7873, 4.665) | 1.659 (0.5505, 6.54) | 1.009 (0.09986, 40.77) | 1.024 (0.4687, 2.077) | 1.62 (0.6883, 3.811) | 1.412 (0.6297, 3.198) | 0.3845 (0.1928, 0.6936) | 0.9906 (0.09489, 42.84) | Retigabine_Ezogabine |  |  |  |
| 0.311 (0.04783, 1.24) | 0.2796 (0.03895, 1.543) | 0.1706 (0.009311, 6.819) | 0.166 (0.0283, 0.6265) | 0.2638 (0.04224, 1.027) | 0.2357 (0.0362, 0.9148) | 0.0625 (0.01091, 0.2095) | 0.1726 (0.008714, 6.615) | 0.1643 (0.02572, 0.6933) | Rufinamide |  |  |
| 1.933 (0.5868, 6.19) | 1.736 (0.4443, 7.438) | 1.069 (0.0927, 54.1) | 1.07 (0.3471, 2.687) | 1.663 (0.5347, 4.681) | 1.463 (0.4487, 4.15) | 0.3975 (0.1392, 0.9563) | 1.069 (0.08805, 55.1) | 1.031 (0.3167, 3.187) | 6.239 (1.119, 44.98) | Topiramate |  |
| 2.221 (0.7267, 6.16) | 1.929 (0.5649, 8.74) | 1.243 (0.1026, 50.87) | 1.188 (0.4342, 2.914) | 1.912 (0.6624, 5.003) | 1.659 (0.5809, 4.34) | 0.4481 (0.1768, 0.9985) | 1.215 (0.09291, 49.9) | 1.179 (0.3781, 3.303) | 7.059 (1.509, 48.18) | 1.129 (0.3205, 4.362) | Zonisamide |

**Table S3.** Pairwise comparison of treatments associated with ≥75% responder rate

Comparisons should be read from left to right. The efficacy estimate is located at the intersection of the column-defining treatment and the row-defining treatment. An RR above 1 favours the column-defining treatment. To obtain RRs for comparisons in the opposing direction, reciprocals should be taken.

| Brivaracetam |  |  |  |  |  |  |  |  |  |  |  |  |  |  |  |
| --- | --- | --- | --- | --- | --- | --- | --- | --- | --- | --- | --- | --- | --- | --- | --- |
| 0.374 (0.01043, 4.786) | Cenobamate |  |  |  |  |  |  |  |  |  |  |  |  |  |  |
| 3.991 (0.9702, 30.8) | 10.71 (1.564, 323.9) | Eslicarbazepine_acetate |  |  |  |  |  |  |  |  |  |  |  |  |  |
| 0.809 (0.0253, 11.52) | 2.176 (0.06021, 78.9) | 0.1991 (0.007434, 1.421) | Gabapentin |  |  |  |  |  |  |  |  |  |  |  |  |
| 1.823 (0.2621, 14.57) | 4.835 (0.5356, 142.7) | 0.4545 (0.09576, 1.533) | 2.17 (0.2316, 66.71) | Lacosamide |  |  |  |  |  |  |  |  |  |  |  |
| 2.928 (0.532, 21.69) | 7.699 (0.9941, 220.7) | 0.7025 (0.2109, 2.137) | 3.432 (0.4125, 105.1) | 1.551 (0.3791, 7.987) | Lamotrigine |  |  |  |  |  |  |  |  |  |  |
| 1.707 (0.3911, 11.94) | 4.476 (0.6541, 132.7) | 0.4209 (0.1566, 1.093) | 2.045 (0.267, 58.2) | 0.9378 (0.2569, 4.275) | 0.5934 (0.1931, 1.931) | Levetiracetam |  |  |  |  |  |  |  |  |  |
| 2.849 (0.536, 21.94) | 7.396 (0.9555, 235.1) | 0.6958 (0.2106, 2.115) | 3.438 (0.4107, 100.7) | 1.56 (0.3614, 8.243) | 1.005 (0.2786, 3.819) | 1.693 (0.4991, 5.159) | Oxcarbazepine |  |  |  |  |  |  |  |  |
| 2.509 (0.4221, 21.48) | 6.828 (0.7329, 215.5) | 0.6118 (0.1579, 2.011) | 3.169 (0.3258, 91.07) | 1.382 (0.2823, 7.028) | 0.8827 (0.1908, 3.311) | 1.475 (0.3718, 4.907) | 0.8842 (0.1791, 3.538) | Perampanel |  |  |  |  |  |  |  |
| 10.99 (2.979, 73.15) | 29.11 (5.169, 794.9) | 2.676 (1.45, 5.487) | 13.33 (2.163, 356.2) | 5.974 (2.196, 24.03) | 3.841 (1.707, 10.4) | 6.441 (3.402, 13.79) | 3.864 (1.603, 10.97) | 4.363 (1.706, 15.1) | Placebo |  |  |  |  |  |  |
| 0.9294 (0.0278, 12.44) | 2.437 (0.06718, 88.68) | 0.2216 (0.007933, 1.546) | 1.113 (0.8153, 1.491) | 0.4996 (0.01766, 4.636) | 0.3197 (0.0107, 2.616) | 0.5394 (0.01916, 3.943) | 0.3206 (0.01037, 2.755) | 0.3516 (0.0123, 3.46) | 0.08224 (0.003029, 0.5203) | Pregabalin |  |  |  |  |  |
| 3.354 (0.3239, 38.74) | 9.341 (0.5758, 353.9) | 0.8377 (0.1077, 4.221) | 4.197 (0.266, 137.5) | 1.877 (0.2096, 14.33) | 1.211 (0.134, 7.155) | 2.022 (0.239, 10.63) | 1.205 (0.1421, 7.417) | 1.355 (0.1379, 9.829) | 0.3156 (0.03927, 1.393) | 3.682 (0.2426, 126.4) | Retigabine_Ezogabine |  |  |  |  |
| 5.491 (0.6336, 50.13) | 14.83 (1.222, 475) | 1.318 (0.2063, 6.062) | 6.528 (0.5409, 221.2) | 2.916 (0.4133, 21.12) | 1.891 (0.2874, 9.853) | 3.187 (0.4873, 13.74) | 1.939 (0.2891, 10.31) | 2.159 (0.3136, 11.92) | 0.4917 (0.09496, 1.858) | 5.956 (0.5037, 201) | 1.609 (0.1602, 15.6) | Rufinamide |  |  |  |
| 3.014 (0.5993, 21.22) | 8.064 (1.011, 237.6) | 0.7254 (0.2327, 2.169) | 3.574 (0.4549, 99.15) | 1.687 (0.4025, 7.89) | 1.061 (0.3009, 3.731) | 1.782 (0.5871, 5.312) | 1.05 (0.2944, 4.214) | 1.159 (0.3312, 5.053) | 0.2737 (0.1095, 0.5723) | 3.212 (0.4139, 93.85) | 0.8829 (0.1534, 7.431) | 0.558 (0.1116, 3.274) | Topiramate |  |  |
| 0.7346 (0.01561, 11.21) | 2.071 (0.04003, 67.18) | 0.1907 (0.00534, 1.356) | 0.9041 (0.02152, 30.22) | 0.4345 (0.01002, 4.239) | 0.2639 (0.005877, 2.221) | 0.4596 (0.01225, 3.288) | 0.2744 (0.006693, 2.333) | 0.3085 (0.007264, 2.854) | 0.07054 (0.001871, 0.4406) | 0.8077 (0.02054, 28.1) | 0.2154 (0.00503, 3.581) | 0.1331 (0.002848, 1.76) | 0.2476 (0.006586, 2.092) | Vigabatrin |  |
| 3.856 (0.6868, 30.78) | 10.63 (1.366, 261.2) | 0.9588 (0.2817, 3.114) | 4.799 (0.5246, 136.4) | 2.192 (0.4391, 11.27) | 1.374 (0.3405, 5.133) | 2.317 (0.6384, 7.791) | 1.407 (0.3664, 5.496) | 1.562 (0.3716, 7.686) | 0.3581 (0.115, 0.9251) | 4.36 (0.4872, 124.5) | 1.165 (0.1733, 10.58) | 0.738 (0.1157, 4.726) | 1.325 (0.3415, 5.033) | 5.171 (0.5299, 224.2) | Zonisamide |

**Table S4.** Pairwise comparison of treatments associated with 100% responder rate

Comparisons should be read from left to right. The efficacy estimate is located at the intersection of the column-defining treatment and the row-defining treatment. An RR above 1 favours the column-defining treatment. To obtain RRs for comparisons in the opposing direction, reciprocals should be taken.

**Table S5.** SUCRA Values of Treatments for Efficacy Outcomes

| **Treatments** | **≥50% responder rate** | **≥75% responder rate** | **100% responder rate** |
| --- | --- | --- | --- |
| Tiagabaine | 0.893088 |  |  |
| Vigabatrin | 0.869412 |  | 0.774533 |
| Valproate | 0.832618 |  |  |
| Cenobamate | 0.813941 | 0.730091 | 0.898267 |
| Pregabalin | 0.811794 | 0.448045 | 0.721467 |
| Gabapentin | 0.802177 | 0.457045 | 0.778167 |
| Lamotrigine | 0.543912 |  | 0.415533 |
| Levetiracetam | 0.488235 | 0.652045 | 0.624567 |
| Zonisamide | 0.415324 | 0.322545 | 0.295133 |
| Topiramate | 0.387059 | 0.388409 | 0.3939 |
| Retigabine_Ezogabine | 0.356471 | 0.385091 | 0.3719 |
| Oxcarbazepine | 0.352441 |  | 0.421133 |
| Eslicarbazepine_acetate | 0.331059 | 0.652091 | 0.2624 |
| Lacosamide | 0.306706 | 0.378955 | 0.586 |
| Rufinamide | 0.303912 | 0.955636 | 0.224267 |
| Brivaracetam | 0.261794 |  | 0.747967 |
| Perampanel | 0.230059 | 0.587136 | 0.4677 |
| Placebo | 0 | 0.042909 | 0.017067 |

| Brivaracetam |  |  |  |  |  |  |  |  |  |  |  |  |  |  |  |  |  |
| --- | --- | --- | --- | --- | --- | --- | --- | --- | --- | --- | --- | --- | --- | --- | --- | --- | --- |
| 1.036 (0.935, 1.135) | Cenobamate |  |  |  |  |  |  |  |  |  |  |  |  |  |  |  |  |
| 1.031 (0.971, 1.094) | 0.997 (0.901, 1.111) | Eslicarbazepine_acetate |  |  |  |  |  |  |  |  |  |  |  |  |  |  |  |
| 1.004 (0.948, 1.061) | 0.97 (0.876, 1.082) | 0.974 (0.912, 1.04) | Gabapentin |  |  |  |  |  |  |  |  |  |  |  |  |  |  |
| 1.046 (0.997, 1.101) | 1.011 (0.919, 1.126) | 1.015 (0.953, 1.086) | 1.042 (0.978, 1.108) | Lacosamide |  |  |  |  |  |  |  |  |  |  |  |  |  |
| 1.019 (0.972, 1.075) | 0.984 (0.896, 1.101) | 0.989 (0.931, 1.055) | 1.015 (0.956, 1.08) | 0.975 (0.922, 1.031) | Lamotrigine |  |  |  |  |  |  |  |  |  |  |  |  |
| 0.978 (0.938, 1.021) | 0.944 (0.864, 1.046) | 0.95 (0.899, 1.007) | 0.975 (0.925, 1.03) | 0.935 (0.89, 0.983) | 0.96 (0.914, 1.006) | Levetiracetam |  |  |  |  |  |  |  |  |  |  |  |
| 1.263 (1.15, 1.392) | 1.22 (1.077, 1.388) | 1.225 (1.104, 1.361) | 1.258 (1.134, 1.395) | 1.207 (1.09, 1.326) | 1.24 (1.117, 1.367) | 1.292 (1.172, 1.419) | Oxcarbazepine |  |  |  |  |  |  |  |  |  |  |
| 1.01 (0.963, 1.061) | 0.974 (0.889, 1.081) | 0.98 (0.921, 1.041) | 1.006 (0.949, 1.066) | 0.964 (0.913, 1.018) | 0.99 (0.936, 1.042) | 1.031 (0.985, 1.079) | 0.8 (0.725, 0.88) | Perampanel |  |  |  |  |  |  |  |  |  |
| 0.967 (0.937, 0.998) | 0.933 (0.857, 1.029) | 0.938 (0.894, 0.985) | 0.963 (0.916, 1.009) | 0.924 (0.886, 0.961) | 0.949 (0.91, 0.985) | 0.988 (0.96, 1.016) | 0.765 (0.7, 0.838) | 0.958 (0.925, 0.993) | Placebo |  |  |  |  |  |  |  |  |
| 1.004 (0.958, 1.05) | 0.969 (0.88, 1.074) | 0.973 (0.92, 1.029) | 0.999 (0.947, 1.053) | 0.959 (0.91, 1.011) | 0.984 (0.933, 1.035) | 1.025 (0.984, 1.069) | 0.793 (0.723, 0.88) | 0.994 (0.946, 1.044) | 1.038 (1.003, 1.073) | Pregabalin |  |  |  |  |  |  |  |
| 1.142 (1.069, 1.222) | 1.103 (0.994, 1.234) | 1.109 (1.029, 1.195) | 1.138 (1.052, 1.226) | 1.09 (1.016, 1.175) | 1.121 (1.044, 1.2) | 1.167 (1.095, 1.245) | 0.904 (0.813, 1.01) | 1.131 (1.06, 1.214) | 1.181 (1.115, 1.251) | 1.138 (1.062, 1.221) | Retigabine_Ezogabine |  |  |  |  |  |  |
| 1.012 (0.959, 1.071) | 0.977 (0.887, 1.094) | 0.982 (0.921, 1.054) | 1.007 (0.943, 1.078) | 0.968 (0.911, 1.03) | 0.992 (0.937, 1.054) | 1.034 (0.981, 1.092) | 0.801 (0.725, 0.894) | 1.003 (0.949, 1.063) | 1.046 (1.002, 1.098) | 1.009 (0.955, 1.07) | 0.886 (0.825, 0.955) | Rufinamide |  |  |  |  |  |
| 1.069 (1, 1.15) | 1.035 (0.923, 1.163) | 1.038 (0.957, 1.13) | 1.064 (0.986, 1.157) | 1.022 (0.949, 1.104) | 1.047 (0.977, 1.135) | 1.092 (1.021, 1.174) | 0.846 (0.761, 0.951) | 1.059 (0.988, 1.143) | 1.105 (1.04, 1.181) | 1.065 (0.993, 1.149) | 0.936 (0.86, 1.022) | 1.056 (0.977, 1.145) | Tigabine |  |  |  |  |
| 1.021 (0.972, 1.077) | 0.988 (0.895, 1.097) | 0.991 (0.93, 1.058) | 1.017 (0.957, 1.084) | 0.975 (0.923, 1.038) | 1.001 (0.944, 1.062) | 1.043 (0.995, 1.099) | 0.809 (0.733, 0.895) | 1.011 (0.959, 1.072) | 1.056 (1.015, 1.103) | 1.017 (0.966, 1.079) | 0.894 (0.835, 0.96) | 1.009 (0.951, 1.072) | 0.956 (0.883, 1.031) | Topiramate |  |  |  |
| 1.254 (1.009, 1.576) | 1.213 (0.957, 1.533) | 1.215 (0.972, 1.53) | 1.251 (1, 1.564) | 1.197 (0.961, 1.5) | 1.228 (0.981, 1.535) | 1.279 (1.032, 1.608) | 0.992 (0.785, 1.262) | 1.243 (0.993, 1.549) | 1.297 (1.042, 1.623) | 1.248 (1.002, 1.554) | 1.099 (0.878, 1.382) | 1.238 (0.994, 1.564) | 1.172 (0.931, 1.474) | 1.224 (0.983, 1.542) | Valproate |  |  |
| 1.034 (0.969, 1.108) | 0.998 (0.902, 1.121) | 1.003 (0.932, 1.083) | 1.03 (0.958, 1.11) | 0.988 (0.921, 1.066) | 1.014 (0.944, 1.095) | 1.056 (0.994, 1.129) | 0.818 (0.735, 0.915) | 1.023 (0.954, 1.1) | 1.069 (1.012, 1.138) | 1.03 (0.966, 1.105) | 0.905 (0.835, 0.986) | 1.021 (0.948, 1.104) | 0.967 (0.882, 1.058) | 1.013 (0.941, 1.094) | 0.826 (0.665, 1.02) | Vigabatrin |  |
| 1.018 (0.964, 1.082) | 0.982 (0.895, 1.099) | 0.987 (0.923, 1.059) | 1.013 (0.951, 1.087) | 0.972 (0.916, 1.038) | 0.999 (0.941, 1.062) | 1.039 (0.986, 1.104) | 0.806 (0.731, 0.895) | 1.008 (0.951, 1.076) | 1.052 (1.006, 1.109) | 1.014 (0.958, 1.079) | 0.891 (0.827, 0.963) | 1.006 (0.941, 1.073) | 0.953 (0.877, 1.031) | 0.997 (0.937, 1.063) | 0.813 (0.652, 1.012) | 0.985 (0.909, 1.063) | Zonisamide |

**Table S6. Pairwise comparison of treatments associated with patient retention rate**

Comparisons should be read from left to right. The efficacy estimate is located at the intersection of the column-defining treatment and the row-defining treatment. An RR above 1 favours the column-defining treatment. To obtain RRs for comparisons in the opposing direction, reciprocals should be taken.

**Table S7.** SUCRA Values for patient retention rate

| **Treatment** | **SUCRA** |
| --- | --- |
| Oxcarbazepine | 0.97 |
| Valproate | 0.92 |
| Retigabine | 0.889 |
| Tiagabine | 0.743 |
| Lacosamide | 0.662 |
| Vigabatrin | 0.551 |
| Cenobamate | 0.536 |
| Eslicarbazepine | 0.531 |
| Lamotrigine | 0.473 |
| Topiramate | 0.457 |
| Zonisamide | 0.444 |
| Rufinamide | 0.4 |
| Perampanel | 0.368 |
| Gabapentin | 0.321 |
| Pregabalin | 0.317 |
| Brivaracetam | 0.276 |
| Levetiracetam | 0.115 |
| Placebo | 0.026 |

| Brivaracetam |  |  |  |  |  |  |  |  |  |  |  |  |  |  |  |  |
| --- | --- | --- | --- | --- | --- | --- | --- | --- | --- | --- | --- | --- | --- | --- | --- | --- |
| 1.035 (0.8253, 1.288) | Cenobamate |  |  |  |  |  |  |  |  |  |  |  |  |  |  |  |
| 0.8871 (0.7462, 1.053) | 0.8572 (0.7007, 1.058) | Eslicarbazepine |  |  |  |  |  |  |  |  |  |  |  |  |  |  |
| 0.9786 (0.8037, 1.185) | 0.945 (0.7525, 1.187) | 1.103 (0.9201, 1.312) | Retigabine |  |  |  |  |  |  |  |  |  |  |  |  |  |
| 0.8909 (0.7287, 1.093) | 0.8614 (0.6836, 1.096) | 1.006 (0.8299, 1.213) | 0.9118 (0.7405, 1.13) | Gabapentin |  |  |  |  |  |  |  |  |  |  |  |  |
| 1.026 (0.8588, 1.226) | 0.9902 (0.8026, 1.231) | 1.155 (0.9848, 1.354) | 1.048 (0.8761, 1.264) | 1.15 (0.9448, 1.399) | Lacosamide |  |  |  |  |  |  |  |  |  |  |  |
| 1.108 (0.8896, 1.396) | 1.07 (0.8384, 1.389) | 1.249 (1.016, 1.554) | 1.133 (0.9078, 1.439) | 1.244 (0.9838, 1.584) | 1.08 (0.8736, 1.352) | Lamotrigine |  |  |  |  |  |  |  |  |  |  |
| 1.139 (0.9836, 1.323) | 1.1 (0.9176, 1.339) | 1.284 (1.135, 1.457) | 1.164 (1.002, 1.367) | 1.278 (1.083, 1.514) | 1.111 (0.9722, 1.272) | 1.028 (0.8446, 1.243) | Levetiracetam |  |  |  |  |  |  |  |  |  |
| 0.9722 (0.8157, 1.164) | 0.9397 (0.7629, 1.175) | 1.096 (0.9353, 1.289) | 0.9933 (0.8303, 1.207) | 1.09 (0.8985, 1.332) | 0.9481 (0.8036, 1.127) | 0.8774 (0.7032, 1.086) | 0.8532 (0.7471, 0.9787) | Oxcarbazepine |  |  |  |  |  |  |  |  |
| 0.9778 (0.8369, 1.143) | 0.9444 (0.779, 1.153) | 1.102 (0.9599, 1.261) | 0.999 (0.8521, 1.181) | 1.096 (0.9199, 1.31) | 0.9533 (0.8254, 1.101) | 0.8822 (0.7189, 1.073) | 0.858 (0.7727, 0.9503) | 1.006 (0.8662, 1.161) | Perampanel |  |  |  |  |  |  |  |
| 1.143 (1.003, 1.309) | 1.103 (0.9317, 1.328) | 1.288 (1.159, 1.438) | 1.167 (1.021, 1.357) | 1.281 (1.103, 1.502) | 1.114 (0.9936, 1.259) | 1.032 (0.8568, 1.233) | 1.003 (0.9428, 1.07) | 1.175 (1.043, 1.324) | 1.169 (1.08, 1.274) | Placebo |  |  |  |  |  |  |
| 0.9352 (0.7897, 1.103) | 0.9034 (0.739, 1.112) | 1.054 (0.9063, 1.22) | 0.956 (0.8049, 1.14) | 1.048 (0.8727, 1.263) | 0.9114 (0.779, 1.065) | 0.8443 (0.6801, 1.033) | 0.8207 (0.7267, 0.9235) | 0.9618 (0.8193, 1.122) | 0.9572 (0.8391, 1.086) | 0.8188 (0.7365, 0.9018) | Pregabalin |  |  |  |  |  |
| 1.033 (0.8857, 1.219) | 0.9985 (0.825, 1.226) | 1.164 (1.015, 1.344) | 1.056 (0.9001, 1.259) | 1.16 (0.9718, 1.39) | 1.007 (0.8723, 1.174) | 0.9338 (0.7598, 1.137) | 0.9067 (0.8155, 1.014) | 1.063 (0.9165, 1.235) | 1.057 (0.9408, 1.198) | 0.9045 (0.8293, 0.9893) | 1.105 (0.9708, 1.271) | Rufinamide |  |  |  |  |
| 1.032 (0.8359, 1.272) | 0.998 (0.7863, 1.27) | 1.164 (0.9592, 1.41) | 1.054 (0.8545, 1.316) | 1.156 (0.9262, 1.446) | 1.007 (0.8245, 1.228) | 0.932 (0.7223, 1.178) | 0.9061 (0.7603, 1.076) | 1.062 (0.8659, 1.293) | 1.056 (0.8807, 1.263) | 0.904 (0.7675, 1.058) | 1.105 (0.9137, 1.335) | 0.9992 (0.8277, 1.193) | Tigabine |  |  |  |
| 0.8725 (0.6657, 1.139) | 0.8438 (0.6307, 1.129) | 0.9842 (0.759, 1.268) | 0.8931 (0.6814, 1.169) | 0.9801 (0.7392, 1.291) | 0.851 (0.655, 1.101) | 0.7869 (0.5855, 1.052) | 0.7655 (0.6012, 0.9699) | 0.8972 (0.6896, 1.161) | 0.8935 (0.6982, 1.138) | 0.7636 (0.604, 0.9581) | 0.9337 (0.7217, 1.198) | 0.8443 (0.6557, 1.079) | 0.8453 (0.6379, 1.123) | Topiramate |  |  |
| 1.125 (0.9085, 1.396) | 1.088 (0.8549, 1.391) | 1.268 (1.042, 1.545) | 1.149 (0.9302, 1.438) | 1.261 (1.006, 1.586) | 1.097 (0.8965, 1.348) | 1.015 (0.7913, 1.296) | 0.9873 (0.8273, 1.18) | 1.158 (0.9427, 1.416) | 1.151 (0.9568, 1.388) | 0.9847 (0.8323, 1.162) | 1.203 (0.9942, 1.466) | 1.089 (0.8982, 1.312) | 1.09 (0.8659, 1.376) | 1.291 (0.9701, 1.727) | Vigabatrin |  |
| 0.9922 (0.8074, 1.196) | 0.9587 (0.757, 1.194) | 1.119 (0.9278, 1.321) | 1.014 (0.8263, 1.225) | 1.113 (0.8932, 1.365) | 0.9675 (0.7955, 1.151) | 0.8943 (0.6972, 1.117) | 0.8708 (0.7367, 1.004) | 1.021 (0.8355, 1.212) | 1.014 (0.8544, 1.181) | 0.8675 (0.7437, 0.9891) | 1.061 (0.8833, 1.251) | 0.9601 (0.7995, 1.121) | 0.9621 (0.7673, 1.175) | 1.135 (0.8597, 1.481) | 0.8819 (0.6985, 1.084) | Zonisamide |

**Table S8:** Pairwise comparison of treatments associated with any TEAE

Comparisons should be read from left to right. The efficacy estimate is located at the intersection of the column-defining treatment and the row-defining treatment. An RR above 1 favours the column-defining treatment. To obtain RRs for comparisons in the opposing direction, reciprocals should be taken.

**Table S9.** SUCRA Values of Treatments for Safety Outcomes

| **Treatments** | **Any TEAEs** | **Rash** | **CNS related TEAEs** | **Non-CNS related TEAEs** |
| --- | --- | --- | --- | --- |
| Levetiracetam | 0.885067 | - | 0.797357 | 0.923088 |
| Perampanel | 0.3937 | 0.295083 | 0.466647 | 0.248735 |
| Pregabalin | 0.25 | - | 0.431762 | 0.497529 |
| Eslicarbazepine | 0.124989 | 0.611833 | 0.27419853 | 0.029412 |
| Rufinamide | 0.598023 | - | 0.026431 | 0.104853 |
| Cenobamate | 0.582575 | - | 0.443319 | 0.282059 |
| Brivaracetam | 0.477053 | - | 0.554093 | 0.604794 |
| Tiagabine | 0.578598 | 0.405833 | 0.453719 | 0.763676 |
| Oxcarbazepine | 0.380075 | - | 0.776094 | 0.159853 |
| Lacosamide | 0.564031 | - | 0.372151 | 0.538206 |
| Zonisamide | 0.448003 | - | 0.660488 | 0.744088 |
| Gabapentin | 0.160633 | - | 0.519671 | 0.660059 |
| Retigabine | 0.404064 | - | 0.272738 | 0.519971 |
| Lamotrigine | 0.77048 | 0.3605 | 0.295162 | 0.336618 |
| Topiramate | 0.165248 | 0.830833 | 0.443175 | 0.838824 |
| Vigabatrin | 0.816273 | 0.281 | 0.655357 | 0.578441 |
| Valproate |  |  | 0.666147 | 0.200088 |
| Placebo | 0.901186 | 0.714917 | 0.89149 | 0.969706 |

| Eslicarbazepine |  |  |  |  |  |  |
| --- | --- | --- | --- | --- | --- | --- |
| 0.6287 (0.1929, 2.363) | Lamotrigine |  |  |  |  |  |
| 0.5401 (0.1166, 2.426) | 0.8411 (0.2361, 2.633) | Perampanel |  |  |  |  |
| 1.099 (0.3819, 3.645) | 1.71 (0.9909, 3.142) | 2.019 (0.7694, 6.657) | Placebo |  |  |  |
| 0.6452 (0.0943, 4.135) | 1.018 (0.1809, 4.301) | 1.176 (0.1969, 7.167) | 0.5887 (0.123, 2.295) | Tiagabine |  |  |
| 2.644 (0.21, 97.49) | 4.052 (0.3636, 140.3) | 4.999 (0.3728, 159.1) | 2.346 (0.2354, 74.44) | 4.224 (0.2775, 164) | Topiramate |  |
| 0.3935 (0.01652, 4.347) | 0.6685 (0.02699, 5.716) | 0.774 (0.03003, 7.919) | 0.3722 (0.01644, 3.136) | 0.6194 (0.02288, 9.251) | 0.1346 (0.001618, 3.824) | Vigabatrin |

**Table S10:** Pairwise comparison of treatments associated with rash

Comparisons should be read from left to right. The efficacy estimate is located at the intersection of the column-defining treatment and the row-defining treatment. An RR above 1 favours the column-defining treatment. To obtain RRs for comparisons in the opposing direction, reciprocals should be taken.

| Brivaracetam |  |  |  |  |  |  |  |  |  |  |  |  |  |  |  |  |  |
| --- | --- | --- | --- | --- | --- | --- | --- | --- | --- | --- | --- | --- | --- | --- | --- | --- | --- |
| 0.807 (0.1846, 3.505) | Cenobamate |  |  |  |  |  |  |  |  |  |  |  |  |  |  |  |  |
| 0.6365 (0.2387, 1.617) | 0.7828 (0.1881, 3.196) | Eslicarbazepine |  |  |  |  |  |  |  |  |  |  |  |  |  |  |  |
| 0.9474 (0.3525, 2.517) | 1.166 (0.2803, 4.862) | 1.49 (0.6189, 3.673) | Gabapentin |  |  |  |  |  |  |  |  |  |  |  |  |  |  |
| 0.7536 (0.2837, 1.968) | 0.9241 (0.2265, 3.885) | 1.183 (0.4961, 2.926) | 0.795 (0.3201, 1.969) | Lacosamide |  |  |  |  |  |  |  |  |  |  |  |  |  |
| 0.6361 (0.2045, 1.914) | 0.7847 (0.1716, 3.601) | 1.005 (0.3588, 2.849) | 0.6725 (0.2302, 1.971) | 0.847 (0.2933, 2.461) | Lamotrigine |  |  |  |  |  |  |  |  |  |  |  |  |
| 1.468 (0.6121, 3.506) | 1.809 (0.4758, 7.077) | 2.315 (1.083, 5.14) | 1.554 (0.7062, 3.523) | 1.958 (0.8909, 4.357) | 2.316 (0.8881, 6.077) | Levetiracetam |  |  |  |  |  |  |  |  |  |  |  |
| 1.51 (0.4769, 4.711) | 1.862 (0.4001, 8.642) | 2.368 (0.8326, 7.067) | 1.593 (0.5342, 4.826) | 2.009 (0.6763, 5.943) | 2.363 (0.7048, 8.074) | 1.022 (0.3765, 2.78) | Oxcarbazepine |  |  |  |  |  |  |  |  |  |  |
| 0.8739 (0.3066, 2.395) | 1.071 (0.2484, 4.672) | 1.366 (0.5379, 3.555) | 0.9209 (0.3453, 2.438) | 1.161 (0.436, 3.009) | 1.368 (0.4445, 4.174) | 0.5922 (0.2462, 1.403) | 0.5772 (0.1817, 1.814) | Perampanel |  |  |  |  |  |  |  |  |  |
| 1.698 (0.8082, 3.529) | 2.09 (0.5919, 7.449) | 2.669 (1.459, 5.013) | 1.792 (0.942, 3.445) | 2.258 (1.191, 4.25) | 2.665 (1.165, 6.209) | 1.153 (0.719, 1.857) | 1.123 (0.4641, 2.721) | 1.951 (0.9468, 4.075) | Placebo |  |  |  |  |  |  |  |  |
| 0.8293 (0.3297, 2.03) | 1.023 (0.2566, 4.012) | 1.311 (0.579, 3.005) | 0.8789 (0.4132, 1.856) | 1.108 (0.473, 2.554) | 1.306 (0.4783, 3.544) | 0.5654 (0.2735, 1.157) | 0.5518 (0.1939, 1.539) | 0.955 (0.3844, 2.354) | 0.4895 (0.2813, 0.837) | Pregabalin |  |  |  |  |  |  |  |
| 0.5978 (0.1859, 1.953) | 0.7372 (0.1542, 3.595) | 0.9437 (0.3189, 2.892) | 0.6357 (0.208, 1.946) | 0.7982 (0.2619, 2.403) | 0.9445 (0.2752, 3.257) | 0.4082 (0.1473, 1.151) | 0.3976 (0.1117, 1.432) | 0.6918 (0.2161, 2.232) | 0.3538 (0.1425, 0.8834) | 0.7207 (0.2513, 2.122) | Retigabine |  |  |  |  |  |  |
| 0.2438 (0.0655, 0.792) | 0.3002 (0.05662, 1.416) | 0.3838 (0.114, 1.154) | 0.2581 (0.07297, 0.7899) | 0.3248 (0.09337, 0.9853) | 0.3831 (0.09652, 1.327) | 0.1661 (0.05164, 0.4658) | 0.1621 (0.03962, 0.5717) | 0.2789 (0.07714, 0.9095) | 0.1438 (0.04968, 0.3625) | 0.2924 (0.08807, 0.8535) | 0.4057 (0.09762, 1.458) | Rufinamide |  |  |  |  |  |
| 0.8438 (0.2622, 2.708) | 1.045 (0.2198, 5.025) | 1.325 (0.4513, 4.111) | 0.8952 (0.2927, 2.762) | 1.119 (0.3696, 3.446) | 1.334 (0.3882, 4.565) | 0.5728 (0.2066, 1.622) | 0.5595 (0.1559, 2.029) | 0.968 (0.3068, 3.153) | 0.4972 (0.2009, 1.241) | 1.016 (0.3563, 2.97) | 1.41 (0.3884, 5.165) | 3.467 (0.9605, 14.46) | Tigabine |  |  |  |  |
| 0.8172 (0.2163, 3.014) | 1.011 (0.1851, 5.332) | 1.29 (0.3716, 4.516) | 0.8679 (0.2416, 3.079) | 1.093 (0.306, 3.828) | 1.291 (0.3216, 5.123) | 0.5586 (0.1682, 1.817) | 0.5444 (0.132, 2.205) | 0.9382 (0.2505, 3.47) | 0.4826 (0.1603, 1.427) | 0.9865 (0.2919, 3.343) | 1.364 (0.3227, 5.556) | 3.375 (0.8176, 15.53) | 0.9706 (0.2312, 3.979) | Topiramate |  |  |  |
| 1.337 (0.2375, 7.607) | 1.642 (0.2202, 12.95) | 2.086 (0.402, 11.57) | 1.406 (0.2605, 7.87) | 1.776 (0.3317, 9.852) | 2.091 (0.3608, 12.45) | 0.9077 (0.1779, 4.72) | 0.8827 (0.1474, 5.465) | 1.533 (0.2782, 8.802) | 0.7834 (0.1656, 3.835) | 1.604 (0.3106, 8.65) | 2.218 (0.363, 13.88) | 5.474 (0.9206, 37.29) | 1.57 (0.2584, 9.851) | 1.629 (0.2409, 11.2) | Valproate |  |  |
| 1.212 (0.367, 3.909) | 1.485 (0.3089, 7.33) | 1.9 (0.6242, 5.945) | 1.274 (0.4051, 4.004) | 1.608 (0.5131, 4.992) | 1.901 (0.542, 6.67) | 0.8214 (0.2855, 2.369) | 0.7983 (0.2187, 2.94) | 1.389 (0.4242, 4.547) | 0.7112 (0.2754, 1.822) | 1.454 (0.4984, 4.357) | 2.009 (0.5362, 7.508) | 4.95 (1.352, 21.08) | 1.416 (0.3886, 5.392) | 1.471 (0.3552, 6.24) | 0.9081 (0.2534, 3.162) | Vigabatrin |  |
| 1.18 (0.4548, 3.062) | 1.456 (0.3583, 5.942) | 1.856 (0.7943, 4.487) | 1.248 (0.5167, 3.04) | 1.568 (0.6517, 3.788) | 1.858 (0.657, 5.264) | 0.8023 (0.3736, 1.738) | 0.7826 (0.2683, 2.276) | 1.353 (0.5314, 3.528) | 0.6947 (0.3816, 1.281) | 1.422 (0.6322, 3.244) | 1.962 (0.6524, 5.913) | 4.84 (1.615, 16.6) | 1.403 (0.4666, 4.164) | 1.439 (0.4202, 5.079) | 0.886 (0.1628, 4.746) | 0.9748 (0.322, 2.978) | Zonisamide |

**Table S11:** Pairwise comparison of treatments associated with CNS related TEAEs

Comparisons should be read from left to right. The efficacy estimate is located at the intersection of the column-defining treatment and the row-defining treatment. An RR above 1 favours the column-defining treatment. To obtain RRs for comparisons in the opposing direction, reciprocals should be taken.

| Brivaracetam |  |  |  |  |  |  |  |  |  |  |  |  |  |  |  |  |  |
| --- | --- | --- | --- | --- | --- | --- | --- | --- | --- | --- | --- | --- | --- | --- | --- | --- | --- |
| 0.7171 (0.3968, 1.396) | Cenobamate |  |  |  |  |  |  |  |  |  |  |  |  |  |  |  |  |
| 0.4777 (0.2774, 0.8702) | 0.6582 (0.4393, 0.9958) | Eslicarbazepine |  |  |  |  |  |  |  |  |  |  |  |  |  |  |  |
| 1.03 (0.601, 1.835) | 1.439 (0.9945, 2.119) | 2.175 (1.614, 2.962) | Gabapentin |  |  |  |  |  |  |  |  |  |  |  |  |  |  |
| 0.9354 (0.5434, 1.633) | 1.297 (0.9007, 1.945) | 1.963 (1.481, 2.66) | 0.9062 (0.6881, 1.193) | Lacosamide |  |  |  |  |  |  |  |  |  |  |  |  |  |
| 0.775 (0.454, 1.403) | 1.076 (0.738, 1.595) | 1.628 (1.207, 2.209) | 0.7518 (0.5608, 0.9846) | 0.8271 (0.6341, 1.076) | Lamotrigine |  |  |  |  |  |  |  |  |  |  |  |  |
| 1.378 (0.8186, 2.435) | 1.921 (1.369, 2.783) | 2.894 (2.287, 3.769) | 1.336 (1.063, 1.675) | 1.476 (1.189, 1.845) | 1.785 (1.435, 2.236) | Levetiracetam |  |  |  |  |  |  |  |  |  |  |  |
| 0.6183 (0.3623, 1.125) | 0.8527 (0.5986, 1.257) | 1.29 (0.9799, 1.748) | 0.5982 (0.4556, 0.7851) | 0.6599 (0.5085, 0.8644) | 0.8003 (0.614, 1.036) | 0.4459 (0.3625, 0.5504) | Oxcarbazepine |  |  |  |  |  |  |  |  |  |  |
| 0.6965 (0.4079, 1.231) | 0.9674 (0.6761, 1.444) | 1.461 (1.117, 1.96) | 0.6738 (0.5188, 0.8811) | 0.7462 (0.5751, 0.9615) | 0.903 (0.6968, 1.167) | 0.5055 (0.416, 0.6081) | 1.132 (0.877, 1.438) | Perampanel |  |  |  |  |  |  |  |  |  |
| 1.448 (0.8747, 2.527) | 2.006 (1.471, 2.88) | 3.039 (2.452, 3.906) | 1.402 (1.153, 1.725) | 1.549 (1.282, 1.874) | 1.868 (1.548, 2.273) | 1.048 (0.9409, 1.169) | 2.346 (1.955, 2.851) | 2.077 (1.773, 2.473) | Placebo |  |  |  |  |  |  |  |  |
| 0.9062 (0.5282, 1.602) | 1.254 (0.8574, 1.895) | 1.899 (1.432, 2.611) | 0.8728 (0.6774, 1.123) | 0.9674 (0.7455, 1.264) | 1.168 (0.8928, 1.503) | 0.6549 (0.5352, 0.7977) | 1.458 (1.15, 1.893) | 1.299 (1.015, 1.638) | 0.6232 (0.5216, 0.7485) | Pregabalin |  |  |  |  |  |  |  |
| 0.9258 (0.5413, 1.634) | 1.282 (0.9056, 1.887) | 1.94 (1.469, 2.562) | 0.8922 (0.6892, 1.157) | 0.9903 (0.773, 1.259) | 1.194 (0.926, 1.529) | 0.6695 (0.546, 0.8107) | 1.495 (1.165, 1.922) | 1.324 (1.044, 1.665) | 0.6369 (0.5403, 0.7446) | 1.021 (0.8055, 1.311) | Retigabine |  |  |  |  |  |  |
| 0.5638 (0.3196, 1.023) | 0.7797 (0.5271, 1.194) | 1.184 (0.8644, 1.649) | 0.5417 (0.4001, 0.7485) | 0.5994 (0.4471, 0.8068) | 0.7268 (0.5441, 0.9758) | 0.4054 (0.3147, 0.5292) | 0.9129 (0.68, 1.217) | 0.8068 (0.6116, 1.078) | 0.3881 (0.3077, 0.4941) | 0.6247 (0.457, 0.8402) | 0.6098 (0.463, 0.8058) | Rufinamide |  |  |  |  |  |
| 1.134 (0.6534, 2.032) | 1.567 (1.084, 2.325) | 2.383 (1.784, 3.183) | 1.095 (0.8358, 1.415) | 1.212 (0.9316, 1.555) | 1.466 (1.136, 1.887) | 0.8223 (0.6683, 0.9926) | 1.836 (1.418, 2.349) | 1.63 (1.264, 2.047) | 0.7851 (0.6506, 0.9196) | 1.258 (0.9713, 1.601) | 1.234 (0.9594, 1.537) | 2.013 (1.499, 2.651) | Tiagabine |  |  |  |  |
| 1.271 (0.6849, 2.432) | 1.761 (1.058, 2.895) | 2.67 (1.731, 4.103) | 1.237 (0.7932, 1.838) | 1.363 (0.8724, 2.01) | 1.652 (1.073, 2.455) | 0.9231 (0.6109, 1.329) | 2.064 (1.351, 3.094) | 1.826 (1.196, 2.67) | 0.8812 (0.5957, 1.237) | 1.407 (0.9159, 2.063) | 1.38 (0.9076, 2.025) | 2.249 (1.449, 3.463) | 1.126 (0.7271, 1.666) | Topiramate |  |  |  |
| 0.5995 (0.2467, 1.448) | 0.8234 (0.3689, 1.793) | 1.255 (0.5809, 2.532) | 0.5786 (0.2646, 1.156) | 0.6402 (0.2921, 1.255) | 0.7773 (0.3522, 1.558) | 0.4374 (0.201, 0.847) | 0.9728 (0.4423, 1.92) | 0.8635 (0.4057, 1.735) | 0.4142 (0.1928, 0.8036) | 0.6677 (0.301, 1.328) | 0.6503 (0.3024, 1.298) | 1.072 (0.4829, 2.185) | 0.5313 (0.2415, 1.047) | 0.4699 (0.2068, 1.033) | Valproate |  |  |
| 0.9633 (0.5656, 1.747) | 1.338 (0.9239, 2.032) | 2.031 (1.481, 2.758) | 0.9345 (0.6927, 1.244) | 1.034 (0.7793, 1.374) | 1.244 (0.9309, 1.658) | 0.6986 (0.5447, 0.8807) | 1.565 (1.188, 2.084) | 1.386 (1.068, 1.8) | 0.6667 (0.5344, 0.8176) | 1.071 (0.803, 1.419) | 1.043 (0.7883, 1.359) | 1.712 (1.244, 2.326) | 0.8532 (0.6492, 1.118) | 0.7614 (0.5053, 1.174) | 1.621 (0.8575, 3.309) | Vigabatrin |  |
| 1.111 (0.6477, 1.95) | 1.532 (1.086, 2.25) | 2.324 (1.782, 3.098) | 1.069 (0.8377, 1.374) | 1.183 (0.9376, 1.506) | 1.433 (1.117, 1.84) | 0.8007 (0.6656, 0.9613) | 1.795 (1.428, 2.277) | 1.583 (1.286, 2.006) | 0.7639 (0.6555, 0.8816) | 1.224 (0.9548, 1.553) | 1.199 (0.9632, 1.499) | 1.967 (1.48, 2.582) | 0.9764 (0.7789, 1.226) | 0.87 (0.6006, 1.327) | 1.85 (0.9267, 4.002) | 1.148 (0.8927, 1.47) | Zonisamide |

**Table S12:** Pairwise comparison of treatments associated with non-CNS related TEAEs

Comparisons should be read from left to right. The efficacy estimate is located at the intersection of the column-defining treatment and the row-defining treatment. An RR above 1 favours the column-defining treatment. To obtain RRs for comparisons in the opposing direction, reciprocals should be taken.

**Supplementary figures**


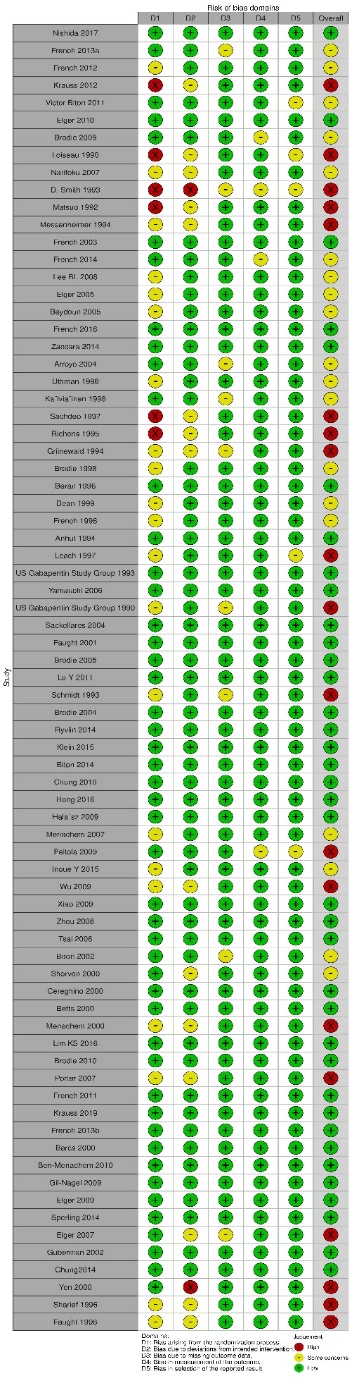


**Figure S1. Summary table for risk of bias for each study**


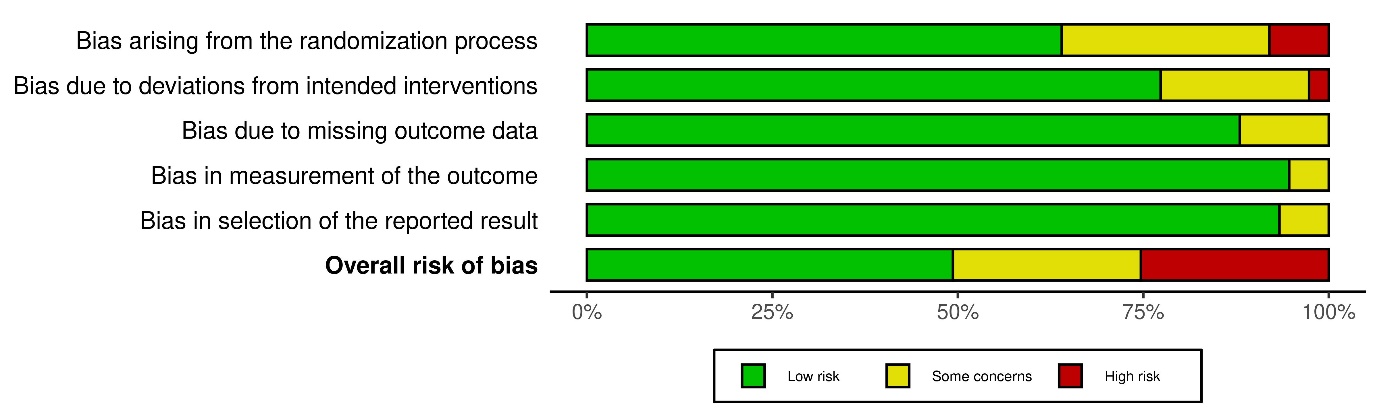


**Figure S2: Visualization of risk of bias**


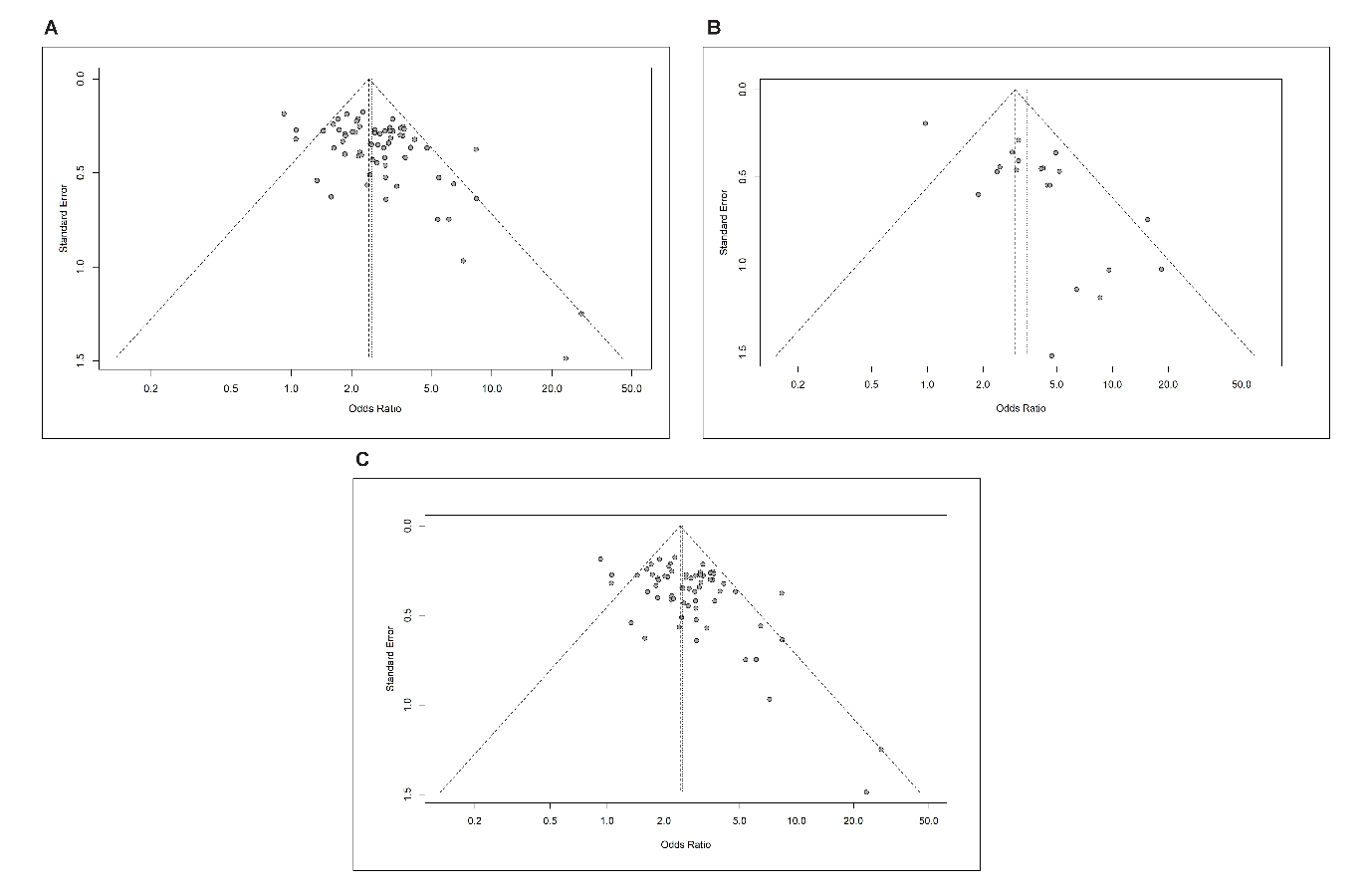


**Figure S3. Publication bias of efficacy outcomes**


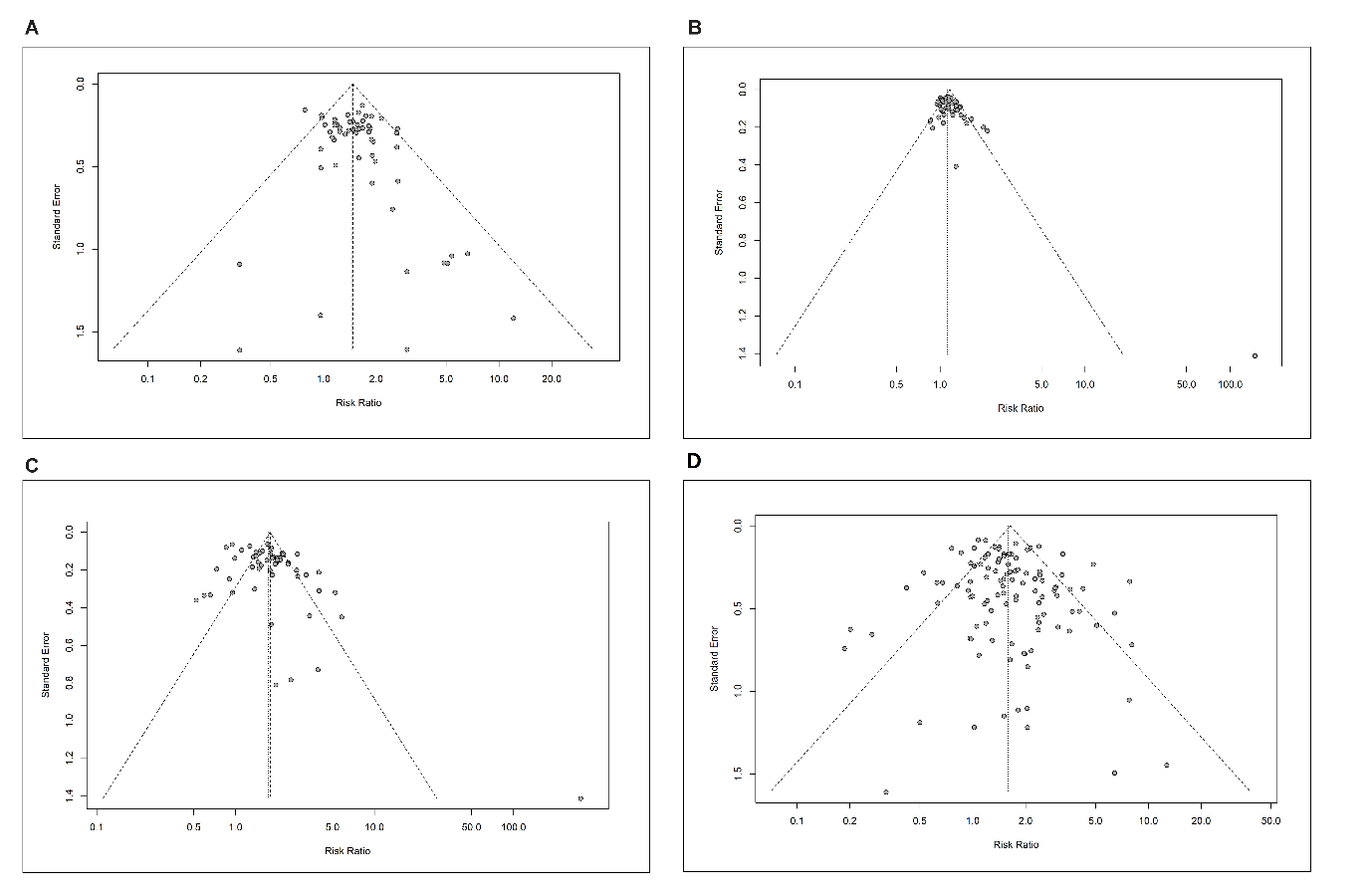


**Figure S4. Publication bias of safety outcomes**

**References**

1. Nishida T, Lee SK, Inoue Y, Saeki K, Ishikawa K, Kaneko S. Adjunctive perampanel in partial-onset seizures: Asia-Pacific, randomized phase III study. Acta Neurol Scand. 2018; 137(4):392–9.

2. French JA, Krauss GL, Steinhoff BJ, Squillacote D, Yang H, Kumar D, et al. Evaluation of adjunctive perampanel in patients with refractory partial-onset seizures: results of randomized global phase III study 305. Epilepsia. 2013; 54(1):117–25.

3. French JA, Krauss GL, Biton V, Squillacote D, Yang H, Laurenza A, et al. Adjunctive perampanel for refractory partial-onset seizures: randomized phase III study 304. Neurology. 2012; 79(6):589–96.

4. Krauss GL, Serratosa JM, Villanueva V, Endziniene M, Hong Z, French J, et al. Randomized phase III study 306: adjunctive perampanel for refractory partial-onset seizures. Neurology. 2012; 78(18):1408–15.

5. Biton V, Krauss G, Vasquez-Santana B, Bibbiani F, Mann A, Perdomo C, et al. A randomized, double-blind, placebo-controlled, parallel-group study of rufinamide as adjunctive therapy for refractory partial-onset seizures. Epilepsia. 2011; 52(2):234–42.

6. Elger CE, Stefan H, Mann A, Narurkar M, Sun Y, Perdomo C. A 24-week multicenter, randomized, double-blind, parallel-group, dose-ranging study of rufinamide in adults and adolescents with inadequately controlled partial seizures. Epilepsy Res. 2010; 88(2–3):255–63.

7. Brodie MJ, Rosenfeld WE, Vazquez B, Sachdeo R, Perdomo C, Mann A, et al. Rufinamide for the adjunctive treatment of partial seizures in adults and adolescents: a randomized placebo-controlled trial. Epilepsia. 2009; 50(8):1899–909.

8. Loiseau P, Yuen AW, Duché B, Ménager T, Arné-Bès MC. A randomised double-blind placebo-controlled crossover add-on trial of lamotrigine in patients with treatment-resistant partial seizures. Epilepsy Res. 1990; 7(2):136–45.

9. Naritoku DK, Warnock CR, Messenheimer JA, Borgohain R, Evers S, Guekht AB, et al. Lamotrigine extended-release as adjunctive therapy for partial seizures. Neurology. 2007; 69(16):1610–8.

10. Smith D, Baker G, Davies G, Dewey M, Chadwick DW. Outcomes of add-on treatment with lamotrigine in partial epilepsy. Epilepsia. 1993; 34(2):312–22.

11. Matsuo F, Bergen D, Faught E, Messenheimer JA, Dren AT, Rudd GD, et al. Placebo-controlled study of the efficacy and safety of lamotrigine in patients with partial seizures. U.S. Lamotrigine Protocol 0.5 Clinical Trial Group. Neurology. 1993; 43(11):2284–91.

12. Messenheimer J, Ramsay RE, Willmore LJ, Leroy RF, Zielinski JJ, Mattson R, et al. Lamotrigine therapy for partial seizures: a multicenter, placebo-controlled, double-blind, cross-over trial. Epilepsia. 1994; 35(1):113–21.

13. French JA, Kugler AR, Robbins JL, Knapp LE, Garofalo EA. Dose-response trial of pregabalin adjunctive therapy in patients with partial seizures. Neurology. 2003; 60(10):1631–7.

14. French J, Brandt C, Friedman D, Biton V, Knapp L, Pitman V, et al. Adjunctive use of controlled-release pregabalin in adults with treatment-resistant partial seizures: a double-blind, randomized, placebo-controlled trial. Epilepsia. 2014; 55(8):1220–8.

15. Lee BI, Yi S, Hong SB, Kim M-K, Lee SA, Lee SK, et al. Pregabalin add-on therapy using a flexible, optimized dose schedule in refractory partial epilepsies: a double-blind, randomized, placebo-controlled, multicenter trial. Epilepsia. 2009; 50(3):464–74.

16. Elger CE, Brodie MJ, Anhut H, Lee CM, Barrett JA. Pregabalin add-on treatment in patients with partial seizures: a novel evaluation of flexible-dose and fixed-dose treatment in a double-blind, placebo-controlled study. Epilepsia. 2005; 46(12):1926–36.

17. Beydoun A, Uthman BM, Kugler AR, Greiner MJ, Knapp LE, Garofalo EA, et al. Safety and efficacy of two pregabalin regimens for add-on treatment of partial epilepsy. Neurology. 2005; 64(3):475–80.

18. French J, Glue P, Friedman D, Almas M, Yardi N, Knapp L, et al. Adjunctive pregabalin vs gabapentin for focal seizures. Neurology. 2016; 87(12):1242–9.

19. Zaccara G, Almas M, Pitman V, Knapp L, Posner H. Efficacy and safety of pregabalin versus levetiracetam as adjunctive therapy in patients with partial seizures: a randomized, double-blind, noninferiority trial. Epilepsia. 2014; 55(7):1048–57.

20. Arroyo S, Anhut H, Kugler AR, Lee CM, Knapp LE, Garofalo EA, et al. Pregabalin add-on treatment: a randomized, double-blind, placebo-controlled, dose-response study in adults with partial seizures. Epilepsia. 2004; 45(1):20–7.

21. Uthman BM, Rowan AJ, Ahmann PA, Leppik IE, Schachter SC, Sommerville KW, et al. Tiagabine for complex partial seizures: a randomized, add-on, dose-response trial. Arch Neurol. 1998; 55(1):56–62.

22. Kälviäinen R, Brodie MJ, Duncan J, Chadwick D, Edwards D, Lyby K. A double-blind, placebo-controlled trial of tiagabine given three-times daily as add-on therapy for refractory partial seizures. Northern European Tiagabine Study Group. Epilepsy Res. 1998; 30(1):31–40.

23. Sachdeo RC, Leroy RF, Krauss GL, Drake ME, Green PM, Leppik IE, et al. Tiagabine therapy for complex partial seizures. A dose-frequency study. The Tiagabine Study Group. Arch Neurol. 1997; 54(5):595–601.

24. Richens A, Chadwick DW, Duncan JS, Dam M, Gram L, Mikkelsen M, et al. Adjunctive treatment of partial seizures with tiagabine: a placebo-controlled trial. Epilepsy Res. 1995; 21(1):37–42.

25. Grünewald RA, Thompson PJ, Corcoran R, Corden Z, Jackson GD, Duncan JS. Effects of vigabatrin on partial seizures and cognitive function. J Neurol Neurosurg Psychiatry. 1994; 57(9):1057–63.

26. Brodie MJ, Mumford JP. Double-blind substitution of vigabatrin and valproate in carbamazepine-resistant partial epilepsy. 012 Study group. Epilepsy Res. 1999; 34(2–3):199–205.

27. Beran RG, Berkovic SF, Buchanan N, Danta G, Mackenzie R, Schapel G, et al. A double-blind, placebo-controlled crossover study of vigabatrin 2 g/day and 3 g/day in uncontrolled partial seizures. Seizure. 1996; 5(4):259–65.

28. Dean C, Mosier M, Penry K. Dose-Response Study of Vigabatrin as add-on therapy in patients with uncontrolled complex partial seizures. Epilepsia. 1999; 40(1):74–82.

29. French JA, Mosier M, Walker S, Sommerville K, Sussman N. A double-blind, placebo-controlled study of vigabatrin three g/day in patients with uncontrolled complex partial seizures. Vigabatrin Protocol 024 Investigative Cohort. Neurology. 1996; 46(1):54–61.

30. Anhut H, Ashman P, Feuerstein TJ, Sauermann W, Saunders M, Schmidt B. Gabapentin (Neurontin) as add-on therapy in patients with partial seizures: a double-blind, placebo-controlled study. The International Gabapentin Study Group. Epilepsia. 1994; 35(4):795–801.

31. Leach JP, Girvan J, Paul A, Brodie MJ. Gabapentin and cognition: a double blind, dose ranging, placebo controlled study in refractory epilepsy. J Neurol Neurosurg Psychiatry. 1997; 62(4):372–6.

32. Gabapentin as add-on therapy in refractory partial epilepsy: a double-blind, placebo-controlled, parallel-group study. The US Gabapentin Study Group No. 5. Neurology. 1993; 43(11):2292–8.

33. Yamauchi T, Kaneko S, Yagi K, Sase S. Treatment of partial seizures with gabapentin: double-blind, placebo-controlled, parallel-group study. Psychiatry Clin Neurosci. 2006; 60(4):507–15.

34. Gabapentin in partial epilepsy. UK Gabapentin Study Group. Lancet Lond Engl. 1990; 335(8698):1114–7.

35. Sackellares JC, Ramsay RE, Wilder BJ, Browne TR, Shellenberger MK. Randomized, controlled clinical trial of zonisamide as adjunctive treatment for refractory partial seizures. Epilepsia. 2004; 45(6):610–7.

36. Faught E, Ayala R, Montouris GG, Leppik IE, Zonisamide 922 Trial Group. Randomized controlled trial of zonisamide for the treatment of refractory partial-onset seizures. Neurology. 2001; 57(10):1774–9.

37. Brodie MJ, Duncan R, Vespignani H, Solyom A, Bitenskyy V, Lucas C. Dose-dependent safety and efficacy of zonisamide: a randomized, double-blind, placebo-controlled study in patients with refractory partial seizures. Epilepsia. 2005; 46(1):31–41.

38. Lu Y, Xiao Z, Yu W, Xiao F, Xiao Z, Hu Y, et al. Efficacy and safety of adjunctive zonisamide in adult patients with refractory partial-onset epilepsy: a randomized, double-blind, placebo-controlled trial. Clin Drug Investig. 2011; 31(4):221–9.

39. Schmidt D, Jacob R, Loiseau P, Deisenhammer E, Klinger D, Despland A, et al. Zonisamide for add-on treatment of refractory partial epilepsy: a European double-blind trial. Epilepsy Res. 1993; 15(1):67–73.

40. Brodie MJ. Zonisamide clinical trials: European experience. Seizure. 2004; 13 Suppl 1:S66-70; discussion S71-72.

41. Ryvlin P, Werhahn KJ, Blaszczyk B, Johnson ME, Lu S. Adjunctive brivaracetam in adults with uncontrolled focal epilepsy: results from a double-blind, randomized, placebo-controlled trial. Epilepsia. 2014; 55(1):47–56.

42. Klein P, Schiemann J, Sperling MR, Whitesides J, Liang W, Stalvey T, et al. A randomized, double-blind, placebo-controlled, multicenter, parallel-group study to evaluate the efficacy and safety of adjunctive brivaracetam in adult patients with uncontrolled partial-onset seizures. Epilepsia. 2015; 56(12):1890–8.

43. Biton V, Berkovic SF, Abou-Khalil B, Sperling MR, Johnson ME, Lu S. Brivaracetam as adjunctive treatment for uncontrolled partial epilepsy in adults: a phase III randomized, double-blind, placebo-controlled trial. Epilepsia. 2014; 55(1):57–66.

44. Chung S, Sperling MR, Biton V, Krauss G, Hebert D, Rudd GD, et al. Lacosamide as adjunctive therapy for partial-onset seizures: a randomized controlled trial. Epilepsia. 2010; 51(6):958–67.

45. Hong Z, Inoue Y, Liao W, Meng H, Wang X, Wang W, et al. Efficacy and safety of adjunctive lacosamide for the treatment of partial-onset seizures in Chinese and Japanese adults: A randomized, double-blind, placebo-controlled study. Epilepsy Res. 2016; 127:267–75.

46. Halász P, Kälviäinen R, Mazurkiewicz-Beldzińska M, Rosenow F, Doty P, Hebert D, et al. Adjunctive lacosamide for partial-onset seizures: Efficacy and safety results from a randomized controlled trial. Epilepsia. 2009; 50(3):443–53.

47. Ben-Menachem E, Biton V, Jatuzis D, Abou-Khalil B, Doty P, Rudd GD. Efficacy and safety of oral lacosamide as adjunctive therapy in adults with partial-onset seizures. Epilepsia. 2007; 48(7):1308–17.

48. Peltola J, Coetzee C, Jiménez F, Litovchenko T, Ramaratnam S, Zaslavaskiy L, et al. Once-daily extended-release levetiracetam as adjunctive treatment of partial-onset seizures in patients with epilepsy: a double-blind, randomized, placebo-controlled trial. Epilepsia. 2009; 50(3):406–14.

49. Inoue Y, Yagi K, Ikeda A, Sasagawa M, Ishida S, Suzuki A, et al. Efficacy and tolerability of levetiracetam as adjunctive therapy in Japanese patients with uncontrolled partial-onset seizures. Psychiatry Clin Neurosci. 2015; 69(10):640–8.

50. Wu X-Y, Hong Z, Wu X, Wu L-W, Wang X-F, Zhou D, et al. Multicenter double-blind, randomized, placebo-controlled trial of levetiracetam as add-on therapy in Chinese patients with refractory partial-onset seizures. Epilepsia. 2009; 50(3):398–405.

51. Xiao Z, Li J-M, Wang X-F, Xiao F, Xi Z-Q, Lv Y, et al. Efficacy and safety of levetiracetam (3,000 mg/Day) as an adjunctive therapy in Chinese patients with refractory partial seizures. Eur Neurol. 2009; 61(4):233–9.

52. Zhou B, Zhang Q, Tian L, Xiao J, Stefan H, Zhou D. Effects of levetiracetam as an add-on therapy on cognitive function and quality of life in patients with refractory partial seizures. Epilepsy Behav EB. 2008; 12(2):305–10.

53. Tsai J-J, Yen D-J, Hsih M-S, Chen S-S, Hiersemenzel R, Edrich P, et al. Efficacy and safety of levetiracetam (up to 2000 mg/day) in Taiwanese patients with refractory partial seizures: a multicenter, randomized, double-blind, placebo-controlled study. Epilepsia. 2006; 47(1):72–81.

54. Boon P, Chauvel P, Pohlmann-Eden B, Otoul C, Wroe S. Dose-response effect of levetiracetam 1000 and 2000 mg/day in partial epilepsy. Epilepsy Res. 2002; 48(1–2):77–89.

55. Shorvon SD, Löwenthal A, Janz D, Bielen E, Loiseau P. Multicenter double-blind, randomized, placebo-controlled trial of levetiracetam as add-on therapy in patients with refractory partial seizures. European Levetiracetam Study Group. Epilepsia. 2000; 41(9):1179–86.

56. Cereghino JJ, Biton V, Abou-Khalil B, Dreifuss F, Gauer LJ, Leppik I. Levetiracetam for partial seizures: results of a double-blind, randomized clinical trial. Neurology. 2000; 55(2):236–42.

57. Betts T, Waegemans T, Crawford P. A multicentre, double-blind, randomized, parallel group study to evaluate the tolerability and efficacy of two oral doses of levetiracetam, 2000 mg daily and 4000 mg daily, without titration in patients with refractory epilepsy. Seizure. 2000; 9(2):80–7.

58. Ben-Menachem E, Falter U. Efficacy and tolerability of levetiracetam 3000 mg/d in patients with refractory partial seizures: a multicenter, double-blind, responder-selected study evaluating monotherapy. European Levetiracetam Study Group. Epilepsia. 2000; 41(10):1276–83.

59. Lim K-S, Lotay N, White R, Kwan P. Efficacy and safety of retigabine/ezogabine as adjunctive therapy in adult Asian patients with drug-resistant partial-onset seizures: A randomized, placebo-controlled Phase III study. Epilepsy Behav EB. 2016; 61:224–30.

60. Brodie MJ, Lerche H, Gil-Nagel A, Elger C, Hall S, Shin P, et al. Efficacy and safety of adjunctive ezogabine (retigabine) in refractory partial epilepsy. Neurology. 2010; 75(20):1817–24.

61. Porter RJ, Partiot A, Sachdeo R, Nohria V, Alves WM, 205 Study Group. Randomized, multicenter, dose-ranging trial of retigabine for partial-onset seizures. Neurology. 2007; 68(15):1197–204.

62. French JA, Abou-Khalil BW, Leroy RF, Yacubian EMT, Shin P, Hall S, et al. Randomized, double-blind, placebo-controlled trial of ezogabine (retigabine) in partial epilepsy. Neurology. 2011; 76(18):1555–63.

63. Krauss GL, Klein P, Brandt C, Lee SK, Milanov I, Milovanovic M, et al. Safety and efficacy of adjunctive cenobamate (YKP3089) in patients with uncontrolled focal seizures: a multicentre, double-blind, randomised, placebo-controlled, dose-response trial. Lancet Neurol. 2020; 19(1):38–48.

64. French JA, Baroldi P, Brittain ST, Johnson JK, PROSPER Investigators Study Group. Efficacy and safety of extended-release oxcarbazepine (Oxtellar XR^TM^) as adjunctive therapy in patients with refractory partial-onset seizures: a randomized controlled trial. Acta Neurol Scand. 2014; 129(3):143–53.

65. Barcs G, Walker EB, Elger CE, Scaramelli A, Stefan H, Sturm Y, et al. Oxcarbazepine placebo-controlled, dose-ranging trial in refractory partial epilepsy. Epilepsia. 2000; 41(12):1597–607.

66. Ben-Menachem E, Gabbai AA, Hufnagel A, Maia J, Almeida L, Soares-da-Silva P. Eslicarbazepine acetate as adjunctive therapy in adult patients with partial epilepsy. Epilepsy Res. 2010; 89(2–3):278–85.

67. Gil-Nagel A, Lopes-Lima J, Almeida L, Maia J, Soares-da-Silva P, BIA-2093-303 Investigators Study Group. Efficacy and safety of 800 and 1200 mg eslicarbazepine acetate as adjunctive treatment in adults with refractory partial-onset seizures. Acta Neurol Scand. 2009; 120(5):281–7.

68. Elger C, Halász P, Maia J, Almeida L, Soares-da-Silva P, BIA-2093-301 Investigators Study Group. Efficacy and safety of eslicarbazepine acetate as adjunctive treatment in adults with refractory partial-onset seizures: a randomized, double-blind, placebo-controlled, parallel-group phase III study. Epilepsia. 2009; 50(3):454–63.

69. Sperling MR, Abou-Khalil B, Harvey J, Rogin JB, Biraben A, Galimberti CA, et al. Eslicarbazepine acetate as adjunctive therapy in patients with uncontrolled partial-onset seizures: Results of a phase III, double-blind, randomized, placebo-controlled trial. Epilepsia. 2015; 56(2):244–53.

70. Elger C, Bialer M, Cramer JA, Maia J, Almeida L, Soares-da-Silva P. Eslicarbazepine acetate: a double-blind, add-on, placebo-controlled exploratory trial in adult patients with partial-onset seizures. Epilepsia. 2007; 48(3):497–504.

71. Guberman A, Neto W, Gassmann-Mayer C, EPAJ-119 Study Group. Low-dose topiramate in adults with treatment-resistant partial-onset seizures. Acta Neurol Scand. 2002; 106(4):183–9.

72. Chung SS, Fakhoury TA, Hogan RE, Nagaraddi VN, Blatt I, Lawson B, et al. Once-daily USL255 as adjunctive treatment of partial-onset seizures: Randomized phase III study. Epilepsia. 2014; 55(7):1077–87.

73. Yen DJ, Yu HY, Guo YC, Chen C, Yiu CH, Su MS. A double-blind, placebo-controlled study of topiramate in adult patients with refractory partial epilepsy. Epilepsia. 2000; 41(9):1162–6.

74. Sharief M, Viteri C, Ben-Menachem E, Weber M, Reife R, Pledger G, et al. Double-blind, placebo-controlled study of topiramate in patients with refractory partial epilepsy. Epilepsy Res. 1996; 25(3):217–24.

75. Faught E, Wilder BJ, Ramsay RE, Reife RA, Kramer LD, Pledger GW, et al. Topiramate placebo-controlled dose-ranging trial in refractory partial epilepsy using 200-, 400-, and 600-mg daily dosages. Topiramate YD Study Group. Neurology. 1996; 46(6):1684–90.
